# SOXE neofunctionalization and elaboration of the neural crest during chordate evolution

**DOI:** 10.1038/srep34964

**Published:** 2016-10-13

**Authors:** Andrew Tai, Martin Cheung, Yong-Heng Huang, Ralf Jauch, Marianne E. Bronner, Kathryn S. E. Cheah

**Affiliations:** 1 School of Biomedical Sciences, Li Ka Shing Faculty of Medicine, The University of Hong Kong, Hong Kong, China; 2 Genome Regulation Laboratory, Drug Discovery Pipeline, Key Laboratory of Regenerative Biology, Guangdong Provincial Key Laboratory of Stem Cell and Regenerative Medicine, South China Institute for Stem Cell Biology and Regenerative Medicine, Guangzhou Institutes of Biomedicine and Health, Chinese Academy of Sciences, Guangzhou, Guangdong, 510530, China; 3 Division of Biology 139-74, California Institute of Technology, Pasadena, USA.

## Abstract

During chordate evolution, two genome-wide duplications facilitated acquisition of vertebrate traits, including emergence of neural crest cells (NCCs), in which neofunctionalization of the duplicated genes are thought to have facilitated development of craniofacial structures and the peripheral nervous system. How these duplicated genes evolve and acquire the ability to specify NC and their derivatives are largely unknown. Vertebrate *SoxE* paralogues, most notably *Sox9/10*, are essential for NC induction, delamination and lineage specification. In contrast, the basal chordate, amphioxus, has a single *SoxE* gene and lacks NC-like cells. Here, we test the hypothesis that duplication and divergence of an ancestral *SoxE* gene may have facilitated elaboration of NC lineages. By using an *in vivo* expression assay to compare effects of *AmphiSoxE* and vertebrate *Sox9* on NC development, we demonstrate that all SOXE proteins possess similar DNA binding and homodimerization properties and can induce NCCs. However, AmphiSOXE is less efficient than SOX9 in transactivation activity and in the ability to preferentially promote glial over neuronal fate, a difference that lies within the combined properties of amino terminal and transactivation domains. We propose that acquisition of *AmphiSoxE* expression in the neural plate border led to NCC emergence while duplication and divergence produced advantageous mutations in vertebrate homologues, promoting elaboration of NC traits.

Urochordates (Tunicates), Cephalochordates (Lancelets) and Vertebrates constitute the three extant groups within the chordate lineage. Although they share the fundamental chordate body plan with a dorsal hollow nerve cord, notochord and paired gill arches, vertebrates uniquely possess neural crest cells (NCCs). The neural crest is a multipotent stem cell population that arises from the dorsal region of the neural tube, undergo an epithelial-mesenchymal transition and migrate to distant locations to differentiate into multiple, diverse derivatives, including melanocytes, peripheral ganglia and craniofacial cartilage. Acquisition of the NC is thought to have facilitated a shift in the chordate lineage from filter-feeders to active predators^1^. Deciphering the molecular underpinnings of how NC is evolved as a multipotent migratory population is essential for understanding the evolutionary origin of vertebrates.

The previous discoveries of migratory pigment cells^2^,^3^ and neuronal progenitors with a subset of neural crest-like properties^4^ within the neural plate border together with phylogenetic analysis place tunicates as the sister group of vertebrates^5^. In contrast, amphioxus completely lacks NC-like cells^6^. Tunicates have small genomes with loss of several developmental genes whereas the genome of amphioxus has retained synteny with vertebrate genome^7^,^8^. Thus amphioxus represents an excellent extant surrogate to the chordate ancestor for exploring conservation and divergence of evolutionary mechanism in NC development.

All vertebrates studied to date contain at least two to three paralogs of *SoxE* transcription factors family (*Sox8*, *Sox9* and *Sox10*), which are crucial for NC development. The relative timing of their expression onset and function during NC development differ depending on the species^9^,^10^,^11^,^12^,^13^,^14^,^15^. In birds and mammals, *Sox9* is the first *SoxE* gene to be expressed in the prospective NCCs and is rapidly downregulated as NC migrates further, whereas *Sox10* expression is activated in the premigratory NC domain and maintained in migrating NCCs^13^. In developing chick embryos, SOX9 induces *Sox10* expression and both are sufficient to transform neural tube progenitors into NC identity. In addition, SOX9 cooperates with SNAIL2 to induce ectopic NCCs with epithelial-mesenchymal transition (EMT) properties^16^,^17^. Prolonged *Sox9* expression biased NC to form glial cells but not neurons^13^. However, NCCs are still specified in mouse embryos with germ-line specific deletion of *Sox9* (genetically null) but undergo apoptosis resulting in defects in the formation of trunk NC-derivatives^16^. In contrast, *Sox8* expression precedes that of *Sox9* and *Sox10* in NC progenitors of *Xenopus* embryos and is required for the proper onset of NC specification^18^. However, in mammals, SOX8 appears to be dispensable for NC development^19^, whereas SOX9 is essential for chondrogenic differentiation^13^,^16^,^20^, and SOX10 for specification of glial, melanocyte and enteric lineages^21^,^22^. These studies highlight species-specific differences in the relative importance of SOXE proteins during NC development. In addition, the chondrogenic expression and function of vertebrate *Sox9* is conserved in three *SoxE* paralogs in lampreys (jawless vertebrates)^9^ and probably in the basal chordate amphioxus, which has a single *SoxE* gene (*AmphiSoxE*) with expression in oral skeleton^23^, suggesting an ancestral role of *SoxE* gene in chondrogenesis. Although previous studies demonstrated a large degree of functional similarity between a *SoxE* ortholog from *Drosophila melanogaster*, *Sox100B* and *Sox10* in mouse NC development^24^, whether *SoxE* gene from amphioxus, the closest living relatives of vertebrates also retains function of and/or differs from vertebrate *Sox9* to regulate NC ontogeny is not clear. Molecular analyses show that the neural-plate border region of amphioxus has many transcription factors common to vertebrates but lacks most “neural crest specifier” genes, most notably *AmphiSoxE*. The exception is *AmphiSNAIL* which is expressed in the dorsolateral portion of the amphioxus neural tube, though no neural crest cells arise from this domain^25^. This implies that the redeployment of *AmphiSoxE* gene and/or other ancestral NC-specifiers in the border region may have facilitated emergence of NCCs. Consistent with this, recent studies suggest that such co-option events could have helped to mediate acquisition of NC cis-regulatory sequences in the *AmphiSoxE* gene^23^. In addition, genome wide duplication results in increasing the number of *SoxE* paralogues and their functional diversity in vertebrates. However, it remains to be determined whether the changes in the coding sequence of *SoxE* genes contribute to their neofunctionalization in NC lineage diversification during the chordate evolution. To address whether duplication and divergence of an ancestral *SoxE* gene may have led to novel functions that facilitated emergence of the NC and its traits in vertebrates, we compared the activity of AmphiSOXE with its vertebrate homologue by analyzing their effects on chicken neural crest development as an assay system.

## Results

### Phylogenetic analyses reveal the intermediate state of amphioxus SOXE during evolution

**Figure 1:**
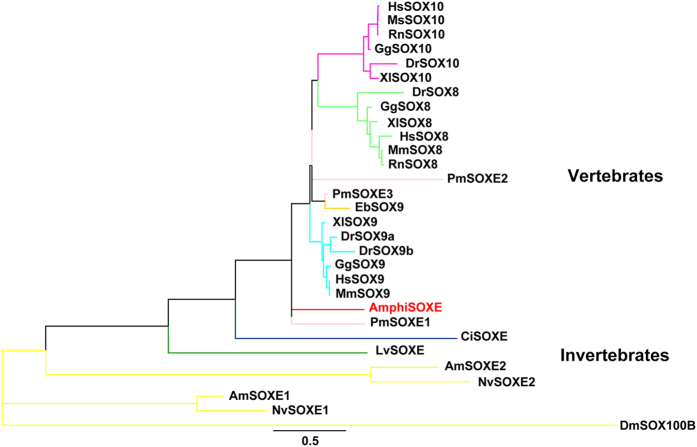
Molecular Phylogenetic analysis of SOXE proteins by Maximum Likelihood method. The evolutionary history was inferred by using the Maximum Likelihood method based on the Dayhoff matrix based model^53^. The tree with the highest log likelihood (−8351.5037) is shown. Initial tree(s) for the heuristic search were obtained automatically by applying Neighbor-Join and BioNJ algorithms to a matrix of pairwise distances estimated using a JTT model, and then selecting the topology with superior log likelihood value. A discrete Gamma distribution was used to model evolutionary rate differences among sites (5 categories (+*G*, parameter = 0.5064)). The tree is drawn to scale, with branch lengths measured in the number of substitutions per site. The analysis involved 30 amino acid sequences. All positions with less than 95% site coverage were eliminated. That is, fewer than 5% alignment gaps, missing data, and ambiguous bases were allowed at any position. There were a total of 273 positions in the final dataset. Evolutionary analyses were conducted in MEGA7^54^,^55^. The trees are exported to Newick files, which is further modified by the figtree without changing the evolutionary distance. *Homo sapiens* (Hs), *Mus musculus* (Mm), *Rattus norvegicus* (Rn), *Gallus gallus* (Gg), *Xenopus laevis* (Xl), *Danio rerio* (Dr), *Petromyzon marinus* (Pm), *Eptatretus burgeri*(Eb), *Branchiostoma lanceolatum*(Amphi), *Ciona intestinalis* (Ci), *Lytechinus variegatus* (Lv), *Drosophila melanogaster* (Dm), *Apis mellifera* (Am), *Nasonia vitripennis* (Nv).

In contrast to vertebrate *SoxE*, *AmphiSoxE* is not expressed in the neural plate border region^26^. Phylogenetic analysis places AmphiSOXE between invertebrates and vertebrates, suggesting its intermediate state during evolution (Fig. 1). Comparative sequence analysis reveals AmphiSOXE shares only 39.1% amino acid identity with Human SOX9 (Supplemental Fig. 1), with highest conservation in the dimerization (71.4%) and HMG (96.2%) domains but only 22% identity in the transactivation domain.

### Amphioxus SOXE and SOX9 show similar DNA binding and cooperative homodimerization

**Figure 2:**
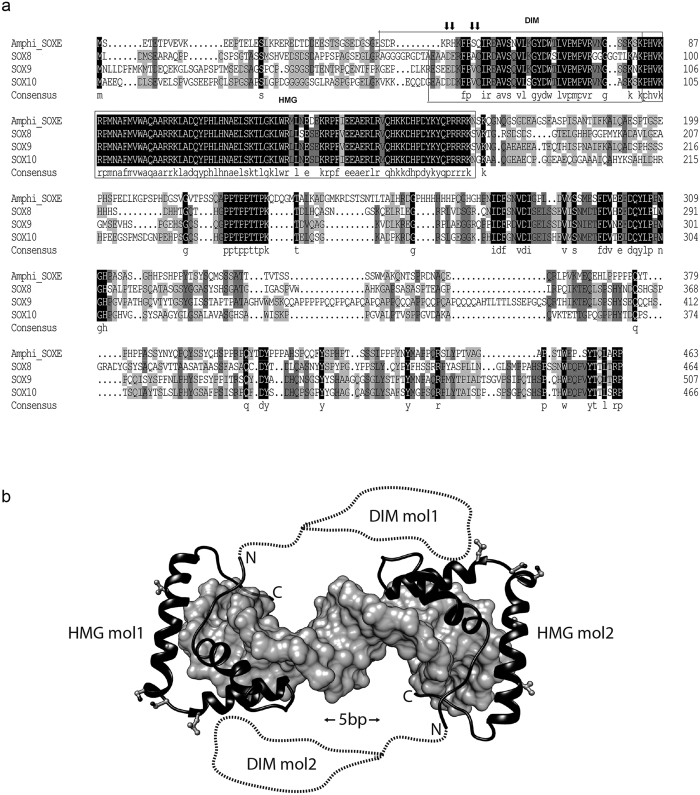
HMG Structure and sequence alignment of SOXE proteins. (**a**) Sequence alignment of SOXE proteins. Invariant residues are shaded black, conserved residues gray and variable residues white. The DIM and the HMG are boxed. The arrows indicate the sites with potential effects on dimerization some of which were interrogated experimentally^28^. (**b**) Structural model of a SOXE dimer on a palindromic DNA sequence with 5bp spacer. Models were prepared as described in Palasingam *et al.*^56^ using structural coordinates from protein data bank entry 3f27 as template (http://www.sciencedirect.com/science/article/pii/S0022283609003635). DNA is shown as gray surface and the HMG boxes of two juxtaposed SOXE molecules as black cartoon. Residues varying amongst HMG boxes of SOXE proteins are shown as ball-and-stick. The DIM domains of unknown structure are schematically depicted with dotted lines.

**Figure 3:**
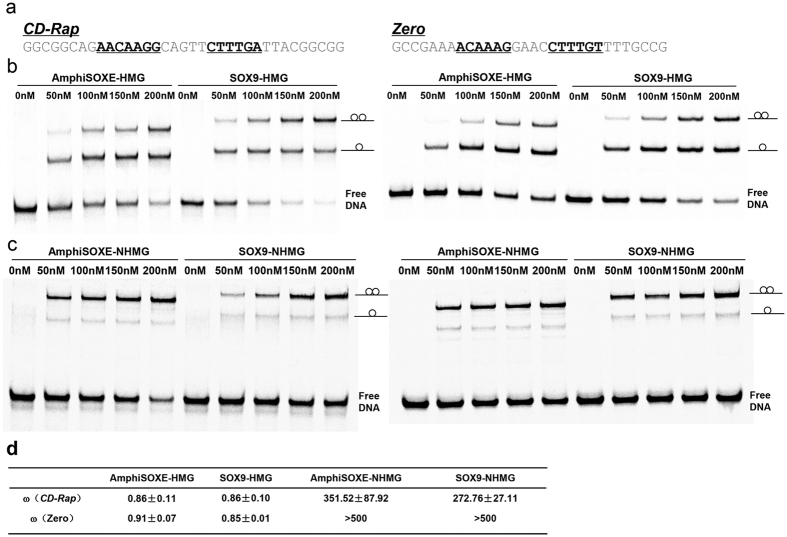
AmphiSOXE and SOX9 show no difference in DNA binding and homodimerization. (**a**) *CD-Rap* (5 bp spacer)^57^ and 4 bp spacer elements used for dimerization EMSA test, the black and bold characters show the core SOX binding sites. (**b,c**) Gel images showing AmphiSOXE and SOX9 binding to the two DNA elements with HMG box constructs (**b**) and NHMG constructs (**c**). (**d**) Cooperativity factors for homodimerization were estimated for the indicted proteins and *CD-Rap* and *Zero* DNA elements. Values are only calculated when the fractional contribution of each of the 3 bands is at least 5%.

Next we asked whether there are detectable changes on the level of SOXE-DNA association that may have contributed to the evolution of new biochemical activities within the SOXE subgroup. AmphiSOXE shares a highly conserved amino acid sequence in both the high-mobility group (HMG) box and the DNA-dependent dimerization (DIM) domain with the mammalian SOXE proteins SOX8, SOX9 and SOX10 (Fig. 2a). The AmphiSOXE HMG box encodes two unique amino acids within the HMG box and a further four amino acids show variability within the SOXE subgroup (Fig. 2a). However, as these residues map to protein interfaces remote from the DNA they are unlikely to directly modulate DNA recognition (Fig. 2b). The N-terminal region upstream of the AmphiSOXE HMG box encodes the 40 amino acid DIM domain characteristic for the SOXE subgroup^27^. The DIM is evolutionarily less conserved than the HMG box and several amino acid variants within the AmphiSOXE-DIM map to positions that were previously reported to influence cooperative homodimerization on palindromic DNA elements^28^. Moreover, SOXE dimerization has recently been suggested to be mediated by DIM:HMG rather than by DIM:DIM interactions^29^ (Fig. 2b). The same study demonstrated that SOXE factors tolerate a flexible half-site spacing and dimeric complexes where observed on all tested elements with spacers ranging from 1 to 10 bp (ACAATG(n_1–10_)CATTGT). However, the cooperativity factor and thus the efficiency of the SOXE homodimerization was found to be highest when the half-spacing was 3, 4 or 5 bp^29^. We therefore compared DNA dependent dimerization of AmphiSOXE to mammalian SOX9 with quantitative electrophoretic mobility shift assays (EMSAs) using two palindromic SOXE binding sites with 4 or 5 bp spacers between the half-sites (Fig. 3a). In the absence of the DIM, the SOX9-HMG as well as the AmphiSOXE-HMG form additive dimers as indicated by a cooperativity factor ω of ~1 (Fig. 3b,d). In contrast, the SOX9-NHMG and AmphiSOXE-NHMG constructs possessing both DIM domain and HMG box dimerize substantially more effectively with strong positive cooperativity on CD-Rap and Zero DNA elements (Fig. 3c,d). However, both SOX9 and AmphiSOXE bound to and dimerized on DNA in an indistinguishable fashion indicating that they exhibit similar capacity for cooperative binding to the same DNA sequence, despite differences in amino acids within DIM and HMG regions.

### Amphioxus SOXE is capable of inducing neural crest-like cells

**Figure 4:**
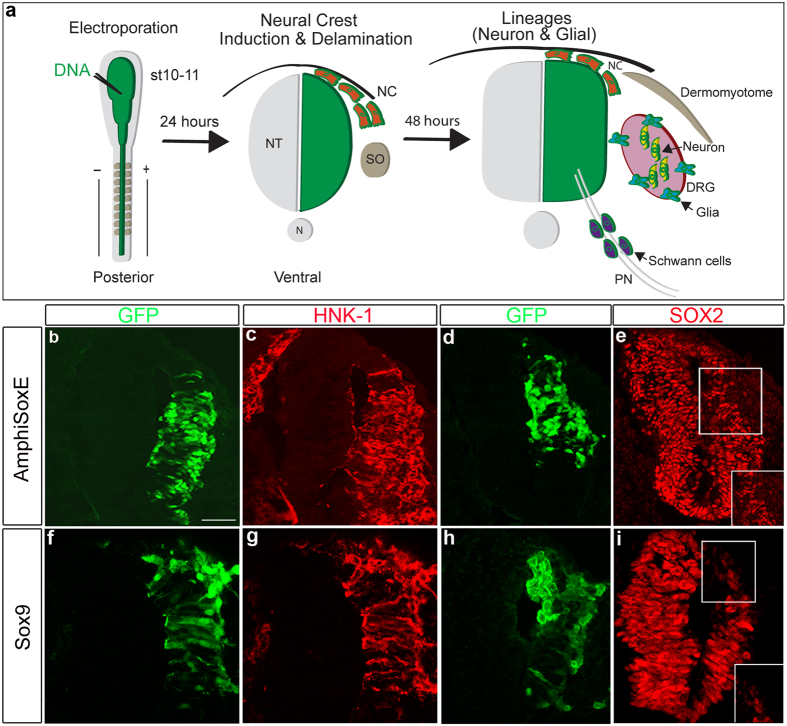
Forced expression of AmphiSOXE induces neural crest-like cells in chick neural tube. (**a**) Schematic representation of the experimental strategy. (**b,f**) Transverse sections of the neural tube electroporated with *AmphiSoxE/EGFP* or *Sox9/EGFP* analysed at 24 hours post-transfection. (**c,g**) Overexpression of *AmphiSoxE* or *Sox9* induces ectopic HNK-1 expression. (**e,i**) Insets show a magnified view of the neural tube region with SOX2 negative cells shown by the white box. The neuronal progenitor marker SOX2 is repressed in (**d,e**) AmphiSOXE or (**h,i**) SOX9 expressing cells in a cell-autonomous manner. Scale bar: 50 μm.

**Figure 5:**
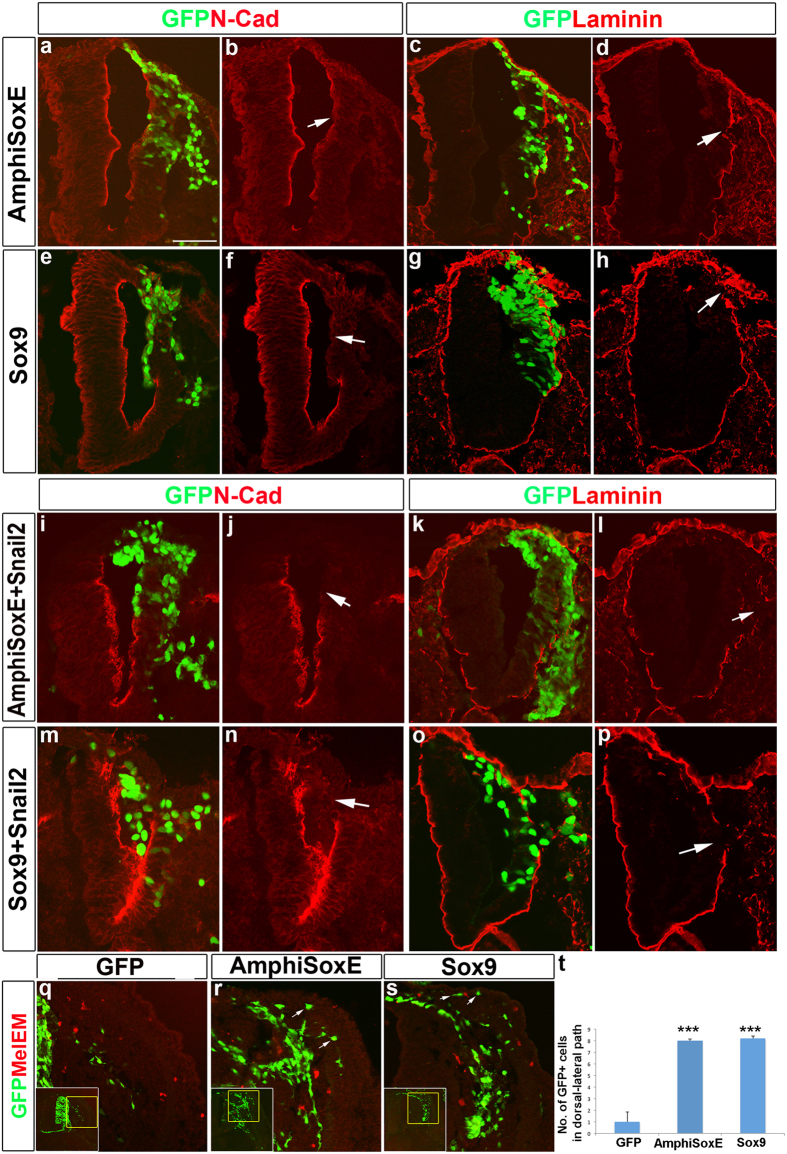
Co-expression of *AmphiSoxE* and *Snail2* causes disruption of apical-basal polarity of neuroepithelial cells. (**a,b,e,f**) Transfection of *AmphiSoxE* or *Sox9* alone: Apical N-CAD expression is reduced as indicated by white arrows. (**c,d,g,h**) Most of the transfected cells are retained in the neural tube except for the dorsal and medial regions where basal LAMININ expression is reduced (white arrows). (**i,j,k,l**) Cotransfection of *AmphiSoxE* and *Snail2* assayed at 24 hours post-transfection. (**i,j**) The apical N-CADHERIN expression is lost (white arrow). (**k,l**) Many cells have delaminated from the neural tube and basal LAMININ expression is lost (white arrow). (**m–p**) A similar observation is seen in the neural tube transfected with *Sox9* and *Snail2*, with loss of N-CAD and LAMININ expression (white arrows). Scale bar: 100 μm. Transverse section of the neural tube electroporated with EGFP (**q**), *AmphiSoxE/EGFP* (**r**) and *Sox9/EGFP* analyzed at 48 hours post-transfection. Early onset of migration onto the dorsolateral migratory pathway is observed in *AmphiSoxE/*EGFP^+^ (**r**) or *Sox9*/EGFP^+^ cells(s) as indicated by the white arrows, but not observed for control EGFP^+^ cells (**q**). These dorsolateral migrating cells do not express the melanocyte marker, MelEM (**r,s**). Insets show a low magnified view of panels q-s with yellow boxes indicating the high magnification of the region where MelEM immunofluorescence is shown in each panel. (**t**) Quantification of the number of EGFP^+^, *AmphiSoxE*/EGFP^+^ and *Sox9*/EGFP^+^ cells migrating via the dorsolateral route. nt, neural tube. ***p < 0.001 as compared to EGFP control. Scale bar: 20 μm.

To compare the activity of AmphiSOXE with its vertebrate homologue, we used an electroporation assay to introduce *AmphiSoxE* or chick *Sox9* cDNA in the bicistronic pCIG nuclear-EGFP expression vector into the caudal neural tube of stage HH10–11 chick embryos prior to neural crest emigration and assessed subsequent effects on NC development (Fig. 4a). After one day (stage HH15), NCCs are actively migrating and by 2 days (HH19) have begun to differentiate into neurons within the ganglion core, whereas bipotential neural and glial precursors remain in the dorsal root ganglia (DRG) periphery^16^. Consistent with the possibility that recruitment of SOXE to the neural plate border may have facilitated emergence of NC, AmphiSOXE promoted NC specification and emigration from the dorsal neural tube similar to SOX9. By 24 hours post-transfection (hpt), like Sox9, AmphiSOXE also induced ectopic expression of markers characteristic of NC identity, including *Sox10*, *FoxD3*, *Wnt3a*, and HNK1, while repressing the neural marker SOX2 (Fig. 4b–i; Supplemental Fig. 2a–f). In contrast, neither ectopic expression of these markers nor alteration of their endogenous levels was observed in the untransfected side of the neural tube or embryos treated with pCIG control vector (Supplemental Figs 2 and 3). As with SOX9^13^, transfected cells delaminated from lateral regions of the neural tube, accompanied by breakdown of LAMININ in the basal lamina (Fig. 5c,d,g,h) and reduction of N-CADHERIN expression in the apical region (Fig. 5a,b,e,f). Like co-expression of *Sox9* and *Snail2*^16^, combined electroporation of *AmphiSoxE* and *Snail2* resulted in ectopic NC emigration due to loss of apical-basal polarity in the transfected cells (Fig. 5i–p).

### Overexpression of Amphioxus SOXE drives both dorsally and ventrally migrating cells toward DRG lineages

**Figure 6:**
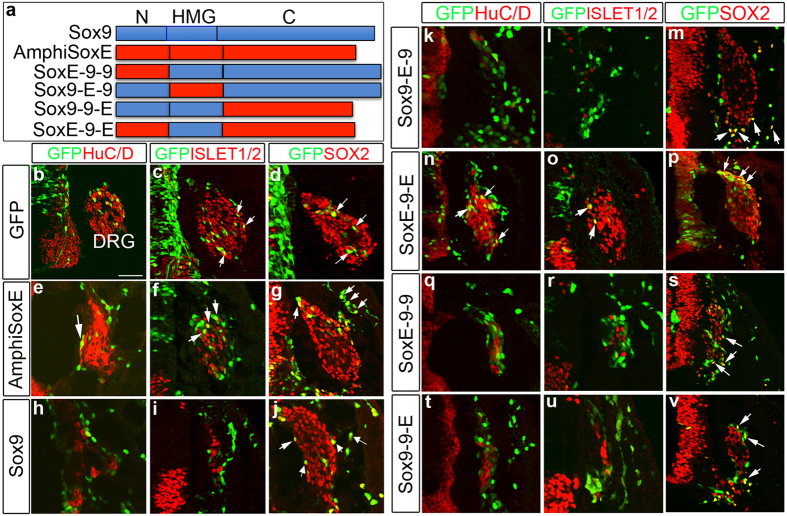
Prolonged expression of *AmphiSoxE* does not affect NC-derived lineages specification at trunk level. (**a**) Schematic diagram of the constructs used for the analysis. Transverse sections of the neural tube electroporated with EGFP (**b–d**), *AmphiSoxE/EGFP* (**e–g**), *Sox9*/EGFP (**h–j**), *Sox9-E-9/*EGFP (**k–m**), *SoxE-9-E*/EGFP (**n–p**), *SoxE-9-9*/EGFP (**q–s**) and *Sox9-9-E*/EGFP (**t–v**) and analysed after 48 hours. (**b–d**) Control EGFP^+^ cells migrate to dorsal root ganglia (DRG) and express markers for neuron, HuC/D and ISLET1/2 and glia, SOX2. *AmphiSoxE*^+^ (**e,f**) and *SoxE-9-E*^+^ cells (**n,o**) express HuC/D and ISLET1/2 in DRG whereas expression of these neuronal markers are lost in embryos transfected with *Sox9* (**h,i**), *Sox9-E-9* (**k,l**), *SoxE-9-9* (**q,r**) and *Sox9-9-E* (**t,u**). SOX2 expression in DRG is unaffected by ectopic expression of each construct (**g,j,m,p,s,v**). The white arrows indicate the transfected cells co-expressing either ISLET1/2 or SOX2. Scale bar: 50 μm.

Despite these similarities at early stages, marked differences were noted between AmphiSOXE, SOX9 and control electroporations at 48hpt. Whereas control electroporations result in GFP^+^ Schwann cell precursors expressing P0 along the ventral roots, cells overexpressing AmphiSOXE or SOX9 still colonized to the ventral root but failed to differentiate into Schwann cells, suggesting that prolonged expression of AmphiSoxE or Sox9 inhibited NC to differentiate into Schwann cell lineage (Supplemental Fig. 4a–c). In addition, we observed premature migration of GFP^+^ cells along the dorsolateral pigment pathway, which normally opens for melanoblast migration only one day later (HH21) in control embryos (Fig. 5q–t). These dorsolaterally located cells fail to express the melanocyte marker MelEM, instead expressing SOX2 (Fig. 5q–s; Fig. 6g,j, n = 10/10), characteristic of sensory neuron specification of NC^30^,^31^, suggesting a cell fate switch from melanocytic to DRG lineages.

Despite these functional similarities, we also noted marked differences in effects mediated by AmphiSOXE versus SOX9. Similar to GFP control, AmphiSOXE overexpressing embryos had GFP^+^ cells expressing early markers of neuronal differentiation HuC/D or ISLET1/2^32^ and SOX2, within the core of the DRG that differentiated into neurons and glial (Fig. 6b–g; Supplemental Fig. 5 a–d’; n = 8/8). By contrast, GFP^+^ cells in SOX9-electroporated embryos did not express neuronal markers in the DRG core, instead localizing to the periphery where glial cells differentiate^30^ (Fig. 6h–j; Supplemental Fig. 5e,f’; n = 12/12). This difference in lineage specification was insensitive to dosage (Supplemental Fig. 6; n = 3/3). Thus, vertebrate SOX9 appears to be functionally divergent from AmphiSOXE in influencing glial versus neuronal differentiation.

### Divergence of SOXE N- and C-terminal domains enabled glial versus neuronal differentiation bias

To examine which protein domains may be responsible for this differential activity, we generated chimeric proteins between the N-terminus, HMG-box and C-terminal transactivation domains of SOX9 and AmphiSOXE and compared their activities (Fig. 6a). N-, HMG or C-terminal domains were designated 9 when derived from SOX9 or E when derived from AmphiSOXE. All the chimeric proteins can induce HNK1 (Supplemental Fig. 7). Each combination (9-E-9; E-9-9; 9-9-E) behaved like SOX9, resulting in differentiation into glial cells but not neurons (Fig. 6k–m, q–s, t–v; Supplemental Fig. 5g,h’, k,l’; m–o’). However, NC expressing chimeric protein containing the SOX9 HMG box with both the N- and C- domains of AmphiSOXE (E-9-E), differentiated into both neurons and glial (Fig. 6n–p; Supplemental Fig. 5i,j’). These results suggest that N-and C-terminal domains of AmphiSOXE but not the HMG box are responsible for the different influence on neuronal and glial differentiation.

### Transactivation activity of AmphiSOXE is weak compared with SOX9

**Figure 7:**
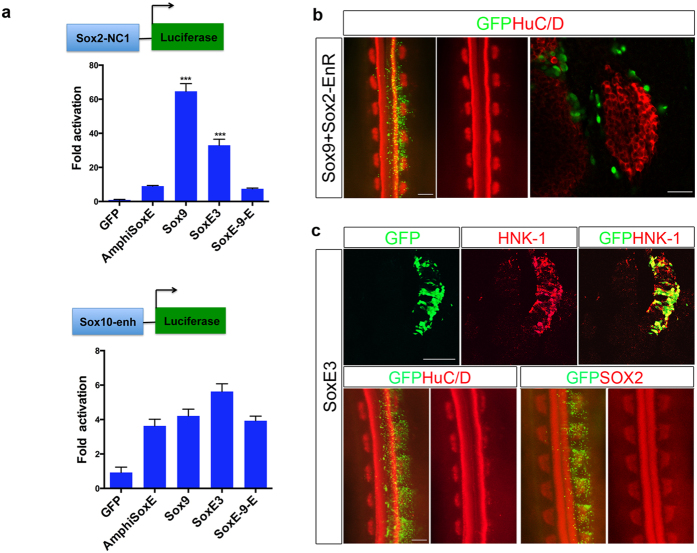
AmphiSOXE is less effective than vertebrate SOX9 in directing *Sox2* transcription. (**a**) In ovo *Sox2-NC1*- and *Sox10* enhancer (*Sox10-enh*) driven luciferase reporter assays: pCAGGS, *AmphiSoxE (SOXE)*, *Sox9*, *SoxE3* and *SoxE-9-E* were cotransfected into the neural tube with a *Sox2-NC1*- or *Sox10-*enhancer (enh)-driven luciferase reporter and a Renilla control plasmid in neural tube. The relative luciferase activity is compared with pCAGGS control. AmphiSOXE or SOXE-9-E protein weakly transactivates the *Sox2-NC1* luciferase reporter whereas SOX9 and SOXE3 yields exceptionally high and moderate activation of the reporter respectively. By contrast, AmphiSOXE, SOX9 or SOXE-9-E exhibits similar transactivation capacities on the *Sox10-enh* luciferase reporter. (**b**) Immunofluorescence on embryos transfected with *Sox9 *+* Sox2-EnR* showing that the transfected cells migrate to the periphery of DRG without affecting HuC/D expression compared to the untransfected side (n = 6). Scale Bar: 50 μm. (**c**) Overexpression of *SoxE3* results in ectopic HNK-1 expression and marked reduction of HuC/D expression in the DRG as compared with the untransfected side, while SOX2 expression remains altered. ***p < 0.001 as compared to AmphiSOXE (SOXE).

Accordingly, we hypothesized that alterations in the ancestral SOXE N-terminus and transactivation domain may have resulted in differences in transactivation activity that could affect lineage decisions. To test this, we compared the transactivation activity of AmphiSOXE with SOX9 *in vivo* using *Sox2*- and *Sox10*-regulatory regions to drive luciferase (*luc*) reporters known to be active in the DRG^33^,^34^. Consistent with the fact that *Sox2* expression is initiated in SOX9^+^ emigrating NCCs^35^ and levels of SOX2 expression are known to influence neuronal differentiation^31^, we found that SOX9 stimulated a marked increase of *Sox2*-*luc* reporter activity compared to AmphiSOXE, SOXE-9-E and vector control (Fig. 7a). These data suggest that AmphiSOXE is a weaker activator of *Sox2* transcription, while SOX9 may inhibit neuronal differentiation by activating high levels of *Sox2* expression (*Sox2*^High^). To test this, we inhibited SOX2 function by overexpressing a *Sox2-EnR* construct^36^ and *Sox9* together and examined the effect on neuronal differentiation in the DRG. By 48hpt, the majority of GFP^+^ cells were localized in the periphery of the DRG rather than the core. Notably DRG size was unchanged and comparable numbers of HuC/D^+^ cells formed in both transfected and control sides (Fig. 7b). These results suggest that blocking SOX2 function in SOX9 overexpressing cells can overcome the differentiation bias in the DRG. In contrast to differences in activation of *Sox2*, we observed similarities in activation of the *Sox10*-*luc* reporter by AmphiSOXE, SOX9 and SOXE-9-E (Fig. 7a), consistent with their similar potency in directing GFP^+^ cells to differentiate into glial.

The basal jawless vertebrate, lamprey (*Petromyzom marinus*) (Fig. 1), has migrating NCCs and most of the NC derivatives. By ectopically expressing the lamprey *Sox9* ortholog, *SoxE3* (Supplemental Fig. 1), we found SoxE3 functions similarly to SOX9, inhibiting sensory neuronal formation without affecting glial differentiation (Fig. 7c). SOXE3 also transactivated *Sox10*-*luc* reporter activity to a similar degree to AmphiSOXE, SOX9 and SOXE-9-E, whereas SOXE3 activated the *Sox2*-*luc* reporter at levels less than for SOX9 but higher than for AmphiSOXE or SOXE-9-E (Fig. 7a). These data suggest that SOXE3 functions similar to SOX9 in directing NC cells to glial rather than neuron differentiation in the DRG.

Lastly, we examined the other two paralogs of vertebrate SOXE, SOX8 and SOX10, which also possessed NC-inducing activity when overexpressed in chick neural tube^13^,^37^. Similar to Sox9, overexpression of SOX8 or SOX10 inhibited neuronal fate without affecting glial cell formation (Supplemental Fig. 8a–l) and exhibited more efficient in transactivating *Sox2-luc* reporter activity than AmphiSOXE (Supplemental Fig. 8m), whereas the activity in transactivating *Sox10-luc* reporter is similar among all tested SOXE proteins (Supplemental Fig. 8n). These findings suggest that divergent function in lineage determination has been fixed and retained in the *SoxE* duplicate paralogs following the split of jawed vertebrates from their ancestors.

## Discussion

**Figure 8:**
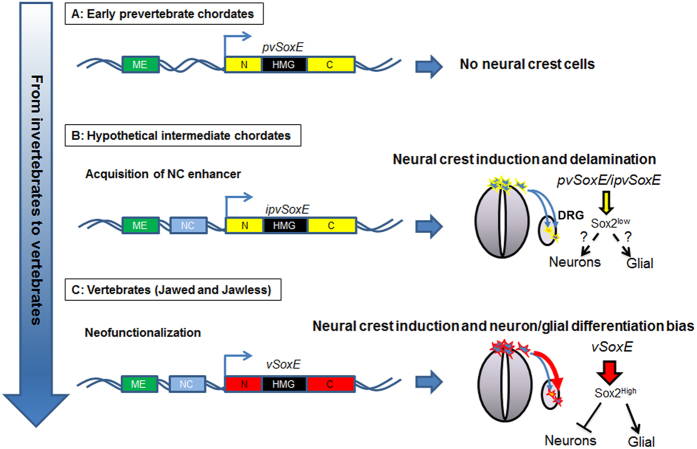
Linking neofunctionalization of SoxE genes to vertebrate neural crest traits. (**A**) An ancient pre-vertebrate chordate (pv-chordate) *pvSoxE* gene is expressed in mesoderm/endoderm under the control of a mesoderm-endodermal enhancer (ME), but lacks a regulatory element(s) that directs expression in the neural plate border and is a weak transactivator. As a consequence the pv-chordate lacks neural crest cells. (**B**) In a hypothetical intermediate pv-chordate/vertebrate *(ipv*), *ipvSoxE* has acquired a neural crest (NC) enhancer, which directs expression in the neural plate border resulting in neural crest formation (blue). However, the ipv SOXE remains as a weak transactivator able to induce low levels of *Sox2* expression without affecting specification of neuronal and glial lineages specification in the DRG. (**C**) Vertebrate Sox9 evolves through alteration of protein sequences in pvSOXE N- and C- terminal domains, which confer with high transactivation capacity to activate high *Sox2* expression resulting in glial fate and suppression of neuronal formation in the DRG.

NC formation is a complex multi-step process^38^, regulated by a hierarchical NC gene regulatory network (NC-GRN) in vertebrates. Studies in lamprey reveal extensive functional conservation of the NC-GRN to the base of vertebrates^39^. In the basal chordate, amphioxus, homologues of vertebrate NC specifier genes are present in the genome but are not expressed at the neural plate border region, with the exception of *AmphiSnail*^26^. Recent data suggest that acquisition of *SoxE* expression in NCCs may have been driven by evolution of new *cis*-regulatory sequences^23^. In addition to regulatory changes, our data suggest that protein changes between AmphiSOXE and vertebrate SOXE may be critical. While AmphiSOXE, SOX9 and even the *Drosophila* ortholog SOX100B can induce NC traits at the expense of neural cell fate^24^, there are clear differences with respect to lineage specification. The domains largely responsible for this differential activity appear to be located in the N- and C-terminus, consistent with the finding that AmphiSOXE is a weaker transactivator of *Sox2* when compared with SOX9 (Fig. 8). Previous studies showed that *Sox2* expression is initiated in early migrating NCCs, maintained in the periphery of the DRG and later restricted to the satellite glial^40^. Overexpression of SOX*2* in trunk NC culture inhibited both neuronal and glial differentiation^40^, whereas downregulation of *Sox2* by shRNA in migrating NCCs or *Wnt1-Cre* mediated knockout of *Sox2* in mouse NC lineage resulted in reduced sensory neurons formation in the DRG^31^. Since SOX2 expression and function was genetically manipulated in uncommitted NC progenitors, the findings from these studies indicate an essential requirement for optimal SOX2 dosage in maintaining bipotent state of NC progenitor in which either excessive or below the optimal level of SOX2 could lead to defects in lineage differentiation. Once NCCs reach the periphery of the DRG, SOX2 and SOX10 are co-expressed in bipotential neural progenitors, which subsequently segregate into SOX2^low^/SOX10^−^ neurons and SOX2^high^/SOX10^+^ glial cells^30^,^31^. This suggests that downregulation of *Sox2* is required for sensory neuron differentiation consistent with its role in the developing chick spinal cord^36^ while SOX2^high^ may promote satellite glial differentiation similar to its function in the oligodendrocyte lineage^41^. In agreement with this, promotion of higher *Sox2* expression by SOX9 compared to AmphiSOXE, correlated with a bias towards glial rather than neuronal differentiation, suggesting that proper levels of SOX2 are required for acquisition of neuronal fate within the DRG. This is further supported by the ability of SOX2-EnR protein to abolish the effect of SOX9 over-expression. Competition between the ectopic SOX2-EnR repressor and the induced endogenous SOX2 for transcriptional targets could lower effective SOX2 transactivation levels to permit neuronal differentiation. Therefore, overexpression of Sox9 exhibited two distinct effects on Sox2 expression at two different stages of NC development, induction (repression of Sox2) and differentiation into glial cells (activation of Sox2) likely through association with stage specific cofactors, which remain to be identified.

As lamprey SOXE3 behaved like SOX9 in promoting glial over neuronal fate, we speculate that the ability of all SOXE proteins to direct NC differentiation into glial may have been acquired in basal vertebrates. In contrast, although another NC specifier, vertebrate *FoxD3*, was able to induce ectopic NCCs when overexpressed in chick neural tube, neither Amphioxus *FoxD* (AmphiFoxD3) nor lamprey *FoxD* paralogs exhibited this activity. Domain mapping studies further revealed that N-terminal sequence is critical for the NCC differentiation-inducing activity of FoxD3^42^. Altogether, these results suggest that co-option of SoxE into NC-GRN occurred before the genome duplication, whereas among the five known vertebrate FoxD paralogs, only FoxD3 is expressed in the NC and might have been recruited slightly later than other NC specifiers, after the genome duplication. In addition, duplicated paralogs of SoxE and FoxD family acquire neofunctionalization properties at the level of NC lineage determination and specification, respectively via changes of specific protein sequences. The reasons underlying the distinct onset of neofunctionalization in both NC specifiers are not clear. Since the C-terminal motif of FoxD3 essential for the differentiation of dorsal mesodermal in *Xenopus* embryos is conserved in AmphiFoxD^42^,^43^, it is tempting to speculate that ancestral FoxD might be important in mesoderm development, resulting in late onset of acquiring the novel ability to induce NCCs.

Our data also imply that acquisition of a stronger transactivation potential in vertebrate SOX9 may have been advantageous during evolution of transcriptional regulation to ensure activation of SOX9 target genes at appropriate levels for tissue development. Consistent with the important contribution of evolving a strongly transactivating SOXE factor for the neural crest in vertebrates, it is noted that heterozygous mutations in the transactivation domain of SOX9 may lead to differential severity of Campomelic Dysplasia because of hypomorphic effects^44^,^45^. It is also notable that some mutations in the transactivation domain of SOX10 can lead to milder Waardenberg syndrome phenotypes than those that cause loss of function^46^,^47^. Our findings indicate that a weaker transactivation activity leads to loss of differential differentiation fate. Thus, we speculate that the two genome-wide duplications in the vertebrate lineage allowed duplication and divergence of *SoxE* genes, conferring additional later functions that may be essential for controlling proper migration and cell lineage segregation. Altogether, our results suggest that acquisition of SOXE to the neural plate border (through a regulatory change) accompanied by duplication and divergence of its protein domains (via advantageous mutations) that strengthened its transactivation ability, allowed elaboration of NC derivatives into peripheral ganglia and perhaps also the craniofacial skeleton (Fig. 8).

## Materials and Methods

All experimental protocols were approved by the Committee on the Use of Live Animals in Teaching and Research (CULATR) in the University of Hong Kong. All the methods were carried out in accordance with the guidelines approved by the Committee on the Use of Live Animals in Teaching and Research (CULATR) in the University of Hong Kong.

### Protein production

The SOX9-HMG and SOX9-NHMG proteins were prepared using a bacterial pET-vector based expression system as described^29^. AmphiSOXE-HMG and the AmphiSOXE-NHMG were PCR amplified using primers with overhangs for attB sites and an N-terminal tobacco etch virus (TEV) protease cleavage site and cloned into a pDEST-hisMBP expression plasmid^48^ using the Gateway BP and LR cloning system (Invitrogen). The expression plasmids were transferred into the BL21(DE3)pLySs *E. coli* strain and the proteins were expressed and purified using established methods^29^.

### EMSA

EMSAs were carried out as reported recently^29^. In brief, dsDNA probes were prepared by combining forward strands with 5′ cy5 label and unlabeled reverse strands (Life Technologies) in 1X annealing buffer (20 mM Tris–HCl, pH 8.0; 50 mM MgCl_2_; 50 mM KCl) followed by heating to 95 °C for 5 min and cooling to 4 °C at 1 °C/min. For each EMSA reaction, 50 nM dsDNA probes were mixed with varying concentrations of protein in 1X EMSA buffer (10 mM Tris–HCl pH 8.0, 0.1 mg/ml bovine serum albumin, 50 μM ZnCl2, 100 mM KCl, 10% (v/v) glycerol, 0.1% (v/v) Igepal CA630 and 2 mM beta-mercaptoethanol). Samples were loaded onto 12% 1X Tris-glycine (TG, 25 mM Tris-HCl pH 8.0, 192 mM glycine) native PAGE gels after incubating at 4 °C in the dark for 4 h. The gel were run at 200 V for 40 min in 1X TG buffer in the cold room and imaged with a Typhoon FLA-7000 PhosphorImager (FUJIFILM). The intensities were detected with the Image Quant software (GE Healthcare) and cooperativity factors were calculated using previously reported equations^49^.

### In ovo electroporation and expression vectors

Fertilized white Leghorn eggs were obtained from Tin Hang Technology Co. Limited and incubated in a humidified incubator at 38 °C. Embryos were staged as described previously^50^. *In ovo* electroporation was carried out as described^13^. Expression constructs were generated in the pCAGGS vector for *mouse Sox8, Sox9, Sox10, AmphiSoxE* and *LampreySoxE3*. Chimeric *SoxE-9-9*, *Sox9-E-9*, *Sox9-9-E* and *SoxE-9-E* conrstructs were generated by DNA recombineering using DY380 bacteria^51^. For electroporation, each *Sox* construct was co-injected with an EGFP expression construct into the lumen of Hamburger Hamilton stage 10–12 neural tubes. Electroporation was carried out using a BTX electroporator delivering five 50-ms pulses of 30V across the neural tube. Transfected embryos were incubated for 24 and 48 h before processing. *Sox2-Luc* reporter was generated by cloning the Sox2-NC1 enhancer^33^ into the pGL3-promoter vector (Promega). *Sox10-Luc* reporter (pE1B-C-F/Sox10-MCS4) was purchased in Addgen (#20243)^34^.Sox2-EnR was a gift of Prof. Jonas Muhr^36^.

### *In situ* hybridisation and immunohistochemistry

Transfected embryos were fixed for an hour at 4 °C in 4% paraformaldehyde in 0.1 M phosphate buffer (PB), cryoprotected with 30% sucrose in PB and cryosectioned. *In situ* hybridization on cryosections was performed as described^13^, using probes for chick*, Sox10*^52^, *Wnt3a* (a gift from N. Itasaki) and *FoxD3* chick expressed sequence tag clone. Immunohistochemical staining was performed using the antibodies against: green fluorescent protein (GFP) (Molecular Probes), Laminin (Sigma), HNK-1 (Becton Dickinson), HuC/D (Invitrogen), SOX2 (Neuromics), Islet1/2 (DSHB), P0 (DSHB) and MelEM (DSHB). Immunofluorescence images were photographed using a Zeiss LSM700 confocal microscope in the Faculty Core Facility, Li Ka Shing Faculty of Medicine, the University of Hong Kong.

### Luciferase assay

*Sox2-NC1* or *Sox10-MCS4* enhancer driven luciferase reporters were mixed with Renilla and the relevant *Sox* expression construct and electroporated into the lumen of the chick neural tube. The trunk parts of embryos were collected 1–2 days later, lysed and assayed following the Promega’s manual. Statistical analysis was performed using a Student’s *t*-test.

## Additional Information

**Accession codes:** Accession numbers for the sequences used in this study are as follows: Rattus norvegicus (Sox8: NP_001100458; Sox10: NP_); Mus musculus (Sox8: NP_035577; Sox9: NM_035578; Sox10: NP_035567); Gallus gallus (Sox8: NP_990062; Sox9: NP_989612; Sox10: NP_990123); Homo sapiens (Sox8: NM_055402; Sox9: NP_000337; Sox10: NP_990123); *Xenopus laevis* (Sox8: NP_001083964; Sox9: NP_001084276; Sox10: NP_001082358); Danio rerio (Sox8: NP_001020636; Sox9a: NP_571718; Sox9b: AAH67133; Sox10: NP_571950); Petromyzon marinus (SoxE1: AAW34332; SoxE2: ABC58684; SoxE3: ABC58685); Eptatretus burgeri (Sox9: BAG11536); Ciona intestinalis (SoxE: CAD58841); Lytechinus variegatus (SoxE: ABY40629); Drosophila melanogaster (Sox100B: NP_651839); Apis mellifera (SoxE1: XP_001122993; SoxE2: XP_001122996) and Nasonia vitripennis (SoxE1: XP_001604913; SoxE2: XP_008213434).

**How to cite this article**: Tai, A. *et al.* SOXE neofunctionalization and elaboration of the neural crest during chordate evolution. *Sci. Rep.* **6**, 34964; doi: 10.1038/srep34964 (2016).

## Supplementary Material

Supplementary Information
