# Supplementary material for: SOXE neofunctionalization and elaboration of the neural crest during chordate evolution
Source: Sci Rep. 2016 Oct 13;6:34964. doi: 10.1038/srep34964 (PMC5062122; doi:10.1038/srep34964)
Supplement: Supplementary Information [file srep34964-s1.pdf]

SOXE neofunctionalization and elaboration of the neural crest during chordate evolution

Andrew Tai[1,4+] Martin Cheung[1,4 +], Yong-Heng Huang[2], Ralf Jauch[2], Marianne E. Bronner[3] and Kathryn S.E. Cheah[1,4*]

[1]Department of Biochemistry, Li Ka Shing Faculty of Medicine, The University of Hong Kong, Hong Kong, China

[2]Genome Regulation Laboratory, Drug Discovery Pipeline, Key Laboratory of Regenerative Biology, Guangdong Provincial Key Laboratory of Stem Cell and Regenerative Medicine, South China Institute for Stem Cell Biology and Regenerative Medicine, Guangzhou Institutes of Biomedicine and Health, Chinese Academy of Sciences, Guangzhou, Guangdong 510530, China [3]Division of Biology 139-74, California Institute of Technology, Pasadena

[4]Present address: School of Biomedical Sciences, Li Ka Shing Faculty of Medicine, The University of Hong Kong, Pokfulam, Hong Kong, China

[+]These authors contributed equally to this work

[*]Corresponding author: kathycheah@hku.hk (KC)

Supplementary Figures and Legends

100%	HsSOX9	1	--
97.1%	MmSOX9	1	--
83.3%	GgSOX9	1	--
69%	DrSOX9a	1	--
54.7%	DrSOX9b	1	--
79.9%	XlSOX9	1	--
45.9%	HsSOX10	1	--
46.4%	MmSOX10	1	--
46.1%	RnSOX10	1	--
46.4%	GgSOX10	1	--
40.8%	DrSOX10	1	--
44.9%	XlSOX10	1	--
44.8%	XlSOX8	1	--
45.8%	GgSOX8	1	--
42.9%	MmSOX8	1	--
42.4%	RnSOX8	1	--
40.8%	HsSOX8	1	--
38.8%	DrSOX8	1	--
33%	PmSOXE1	1	--
32%	PmSOXE2	1	--
53%	PmSOXE3	1	--
50%	EbSOX9	1	--
39.1%	AmphiSOXE	1	--
25.3%	LvSOXE	1	--
18.6%	CiSOXE	1	MNNDMTLLSGHKQTMFNQQSGSQDKWSSVSPNSPDKDIMEHLRKGTDFSCFASSNDVEFS
20.2%	AmSOXE2	1	--
20%	AmSOXE1	1	--
18.8%	DmSOX100B	1	--
19.6%	NvSOXE2	1	--
19.1%	NvSOXE1	1	--

HsSOX9	1	----------------------MNLLDPFMKMTDEQEK-GLSGAPSPTMSED-SAGSPCP
MmSOX9	1	----------------------MNLLDPFMKMTDEQEK-GLSGAPSPTMSED-SAGSPCP
GgSOX9	1	----------------------MNLLDPFMKMTEEQDK-CISDAPSPTMSDD-SAGSPCP
DrSOX9a	1	----------------------MNLLDPYLKMTDEQEK-CLSDAPSPSMSED-SAGSPCP
DrSOX9b	1	-------------------------------MS-------VSGAPSPSLSED-SAGSPCA
XlSOX9	1	----------------------MNLLDPFMKMTEEQDK-CMSGAPSPTMSDD-SAGSPCP
HsSOX10	1	-------------------------------MAEEQD--LSEVELSPVGSEEPRCLSPGS
MmSOX10	1	-------------------------------MAEEQD--LSEVELSPVGSEEPRCLSPGS
RnSOX10	1	-------------------------------MAEEQD--LSEVELSPVGSEEPRCLSPSS
GgSOX10	1	-------------------------------MADDQD--LSEVEMSPVGSEDHHCLSPG-
DrSOX10	1	------------------------------MSAEEHS--MSEVEMSPGVSDDGHSMSPGH
XlSOX10	1	-------------------------------MSDDQS--LSEVEMSPVGSEDP-SLTPDP
XlSOX8	1	----------------------------MLNMSSDQ-----EPPCSP-------TGTASS
GgSOX8	1	----------------------------MLNMTEEHDK-ALEAPCSP-------AGTTSS
MmSOX8	1	----------------------------MLDMSEARA----QPPCSP-------SGTASS
RnSOX8	1	----------------------------MLDMSEARA----QPPCSP-------SGTASS
HsSOX8	1	----------------------------MLDMSEARS----QPPCSP-------SGTASS
DrSOX8	1	-------------------------------MSEER-----EKCSSP-------TGSCSS
PmSOXE1	1	-------------------------------MAEKRLHNMLSHVPSPDVSDVESEPSLHG
PmSOXE2	1	-------------------------------MSDTNHE--MSRTPSPHCSSDAGSGLGSP
PmSOXE3	1	-----------------------------MMSDEQHDK-HMSDVPSPDMSENCSVGSPAD
EbSOX9	1	------------------------------MEDE-----RMSATPSPALSEHSSVGSTVD
AmphiSOXE	1	-------------------------------MSETETP--VEVKEEP-------TELESL
LvSOXE	1	--MSSPESLELHHSLSEGSSPR
CiSOXE	61	SGVEGKENGSKGKSGGPFSPEDLFKPNQDENSTSTSEIFSMSSPESLSNFCNVKSAVAVA
AmSOXE2	1	--MES-------YHHGNDVSGT
AmSOXE1	1	----------------------------MNDIMGEAGSGSISSSATPSVANSANGSTAIN
DmSOX100B	1	--MSDSSSSNCSKDRAKPVETL
NvSOXE2	1	--METNASSCNNNNNNNNNNNN
NvSOXE1	1	---------------------------------MDGTG-ESTKSIALATAAGLNGLVVVN

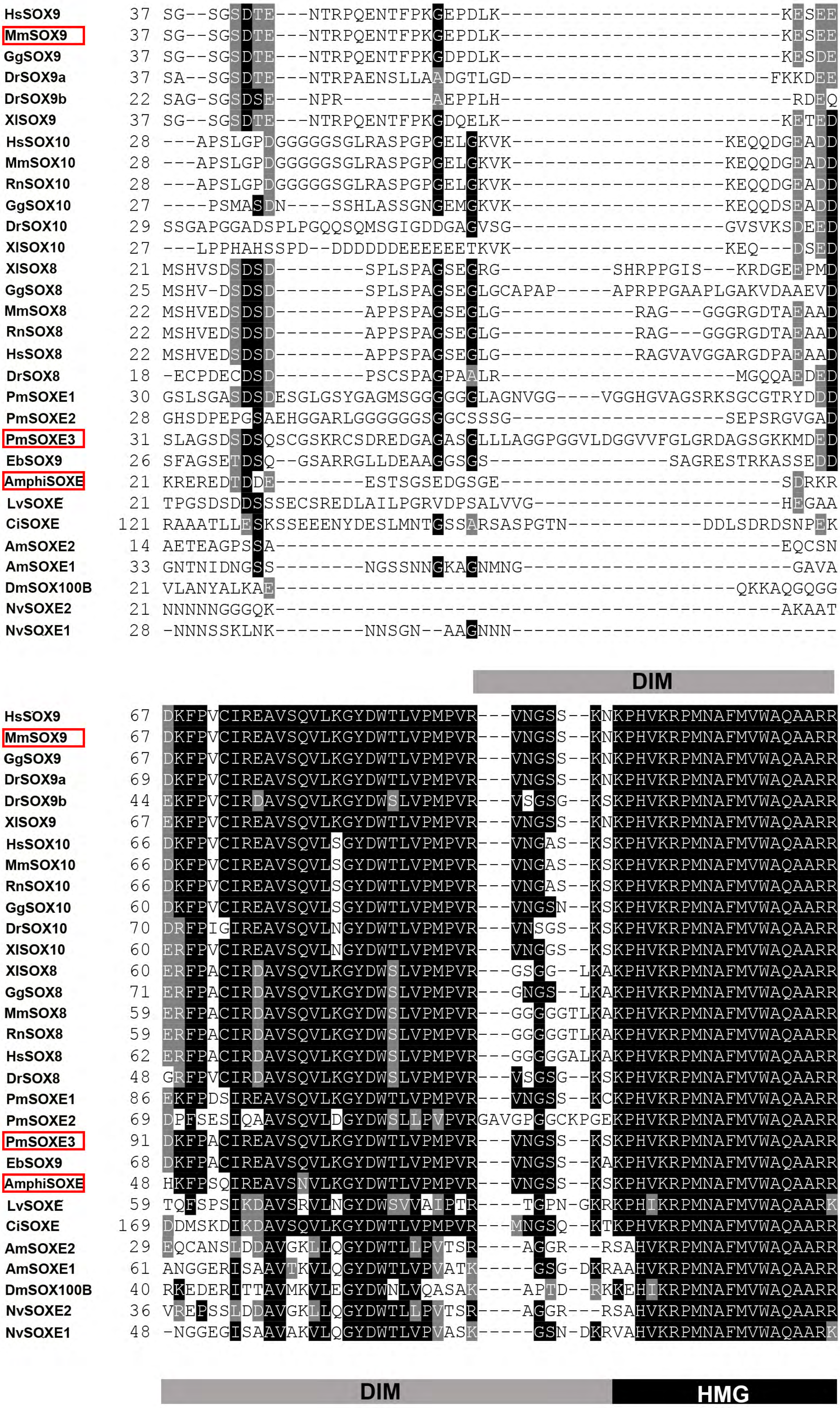
HsSOX9 37 SG--SGSDTE---NTRPQENTFPKGEPDLK------------------------KESEE
MmSOX9 37 SG--SGSDTE---NTRPQENTFPKGEPDLK------------------------KESEE
GgSOX9 37 SG--SGSDTE---NTRPQENTFPKGDPDLK------------------------KESDE
DrSOX9a 37 SA--SGSDTE---NTRPAENSLLAADGTLGD----------------------FKKDEE
DrSOX9b 22 SAG-SGSDSE---NPR--------AEPPLH-------------------------RDEQ
XlSOX9 37 SG--SGSDTE---NTRPQENTFPKGDQELK------------------------KETED
HsSOX10 28 ---APSLGPDGGGGGSGLRASPGPGELGKVK------------------KEQQDGEADD
MmSOX10 28 ---APSLGPDGGGGGSGLRASPGPGELGKVK------------------KEQQDGEADD
RnSOX10 28 ---APSLGPDGGGGGSGLRASPGPGELGKVK------------------KEQQDGEADD
GgSOX10 27 ----PSMASDN----SSHLASSGNGEMGKVK------------------KEQQDSEADD
DrSOX10 29 SSGAPGGADSPLPGQQSQMSGIGDDGAGVSG------------------GVSVKSDEED
XlSOX10 27 ---LPPHAHSSPD--DDDDDDEEEEEETKVK------------------KEQ---DSED
XlSOX8 21 MSHVSDSDSD--------SPLSPAGSEGRG----------SHRPPGIS---KRDGEEPMD
GgSOX8 25 MSHV-DSDSD--------SPLSPAGSEGLGCAPAP-----APRPPGAAPLGAKVDAAEVD
MmSOX8 22 MSHVEDSDSD--------APPSPAGSEGLG------------RAG---GGGRGDTAEAAD
RnSOX8 22 MSHVEDSDSD--------APPSPAGSEGLG------------RAG---GGGRGDTAEAAD
HsSOX8 22 MSHVEDSDSD--------APPSPAGSEGLG------------RAGVAVGGARGDPAEAAD
DrSOX8 18 -ECPDECDSD--------PSCSPAGPAALR--------------------MGQQAEDED
PmSOXE1 30 GSLSGASDSDESGLGSYGAGMSGGGGGLAGNVGG----VGGHGVAGSRKSGCGTRYDDD
PmSOXE2 28 GHSDPEPGSAEHGGARLGGGGGGSGGCSSSG------------------SEPSRGVGAD
PmSOXE3 31 SLAGSDSDSQSCGSKRCSDREDGAGASGLLLAGGPGGVLDGGVVFGLGRDAGSGKKMDED
EbSOX9 26 SFAGSETDSQ--GSARRGLLDEAAGGSGS----------------SAGRESTRKASSEDD
AmphiSOXE 21 KRERED TDDE--------ESTSGSEDGSGE------------------------SDRKR
LvSOXE 21 TPGSDSDDSSSECSREDLAILPGRVDPSALVVG---------------------HEGAA
CiSOXE 121 RAAATLLESKSSEEENYDESLMNTGSSARSASPGTN------------DDLSDRDSNPEK
AmSOXE2 14 AETEAGPSSA--EQCSN
AmSOXE1 33 GNTNIDNGSS--------NGSSNNGKAGNMNG------------------------GAVA
DmSOX100B 21 VLANYALKAE---------------------------------------QKKAQGQGG
NvSOXE2 21 NNNNNGGGQK--AKAAT
NvSOXE1 28 -NNNSSKLNK--------NNSGN--AAGNNN---------------------------
DIM
HsSOX9 67 DKFPVCIREAVSQVLKGYDWTLVPMPVR---VNGSS--KNKPHVKRPMNAFMVWAQAARR
MmSOX9 67 DKFPVCIREAVSQVLKGYDWTLVPMPVR---VNGSS--KNKPHVKRPMNAFMVWAQAARR
GgSOX9 67 DKFPVCIREAVSQVLKGYDWTLVPMPVR---VNGSS--KNKPHVKRPMNAFMVWAQAARR
DrSOX9a 69 DKFPVCIREAVSQVLKGYDWTLVPMPVR---VNGSS--KNKPHVKRPMNAFMVWAQAARR
DrSOX9b 44 EKFPVCIRDAVSQVLKGYDWSLVPMPVR---VSGSG--KSKPHVKRPMNAFMVWAQAARR
XlSOX9 67 EKFPVCIREAVSQVLKGYDWTLVPMPVR---VNGSS--KNKPHVKRPMNAFMVWAQAARR
HsSOX10 66 DKFPVCIREAVSQVLSGYDWTLVPMPVR---VNGAS--KSKPHVKRPMNAFMVWAQAARR
MmSOX10 66 DKFPVCIREAVSQVLSGYDWTLVPMPVR---VNGAS--KSKPHVKRPMNAFMVWAQAARR
RnSOX10 66 DKFPVCIREAVSQVLSGYDWTLVPMPVR---VNGAS--KSKPHVKRPMNAFMVWAQAARR
GgSOX10 60 DKFPVCIREAVSQVLSGYDWTLVPMPVR---VNGSN--KSKPHVKRPMNAFMVWAQAARR
DrSOX10 70 DRFPIGIREAVSQVLNGYDWTLVPMPVR---VNSGS--KSKPHVKRPMNAFMVWAQAARR
XlSOX10 60 ERFPVCIREAVSQVLNGYDWTLVPMPVR---VNGGS--KSKPHVKRPMNAFMVWAQAARR
XlSOX8 60 ERFPACIRDAVSQVLKGYDWSLVPMPVR---GSGG--LKAKPHVKRPMNAFMVWAQAARR
GgSOX8 71 ERFPACIRDAVSQVLKGYDWSLVPMPVR---GNGS--LKAKPHVKRPMNAFMVWAQAARR
MmSOX8 59 ERFPACIRDAVSQVLKGYDWSLVPMPVR---GGGGGTLKAKPHVKRPMNAFMVWAQAARR
RnSOX8 59 ERFPACIRDAVSQVLKGYDWSLVPMPVR---GGGGGTLKAKPHVKRPMNAFMVWAQAARR
HsSOX8 62 ERFPACIRDAVSQVLKGYDWSLVPMPVR---GGGGGALKAKPHVKRPMNAFMVWAQAARR
DrSOX8 48 GRFPVCIRDAVSQVLKGYDWSLVPMPVR---VSGSG--KSKPHVKRPMNAFMVWAQAARR
PmSOXE1 86 EKFPDSIREAVSQVLKGYDWTLVPMPVR---VNGSS--KCKPHVKRPMNAFMVWAQAARR
PmSOXE2 69 DPFSESIQAAVSQVLDGYDWSLLPVPVRGAVGPGGCKPGEKPHVKRPMNAFMVWAQAARR
PmSOXE3 91 DKFPACIREAVSQVLKGYDWTLVPMPVR---VNGSS--KSKPHVKRPMNAFMVWAQAARR
EbSOX9 68 DKFPACIREAVSQVLKGYDWTLVPMPVR---VNGSS--KAKPHVKRPMNAFMVWAQAARR
AmphiSOXE 48 HKFPSQIREAVSNVLKGYDWTLVPMPVR---VNGSS--KSKPHVKRPMNAFMVWAQAARR
LvSOXE 59 TQFSPSIKDAVSRVLNGYDWSVVAIPTR----TGPN-GKRKPHIKRPMNAFMVWAQAARK
CiSOXE 169 DDMSKDIKDAVSQVLKGYDWTLVPMPVR---MNGSQ--KTKPHVKRPMNAFMVWAQAARR
AmSOXE2 29 EQCANSLDDAVGKLLQGYDWTLLPVTSR----AGGR---RSAHVKRPMNAFMVWAQAARR
AmSOXE1 61 ANGGERISAAVTKVLQGYDWTLVPVATK-----GSG-DKRAAHVKRPMNAFMVWAQAARR
DmSOX100B 40 RKEDERITTAVMKVLEGYDWNLVQASAK----APTD--RKKEHIKRPMNAFMVWAQAARR
NvSOXE2 36 VREPSSLDDAVGKLLQGYDWTLLPVTSR----AGGR---RSAHVKRPMNAFMVWAQAARR
NvSOXE1 48 -NGGEGISAAVAKVLQGYDWTLVPVASK-----GSN-DKRVAHVKRPMNAFMVWAQAARK
DIM
HMG

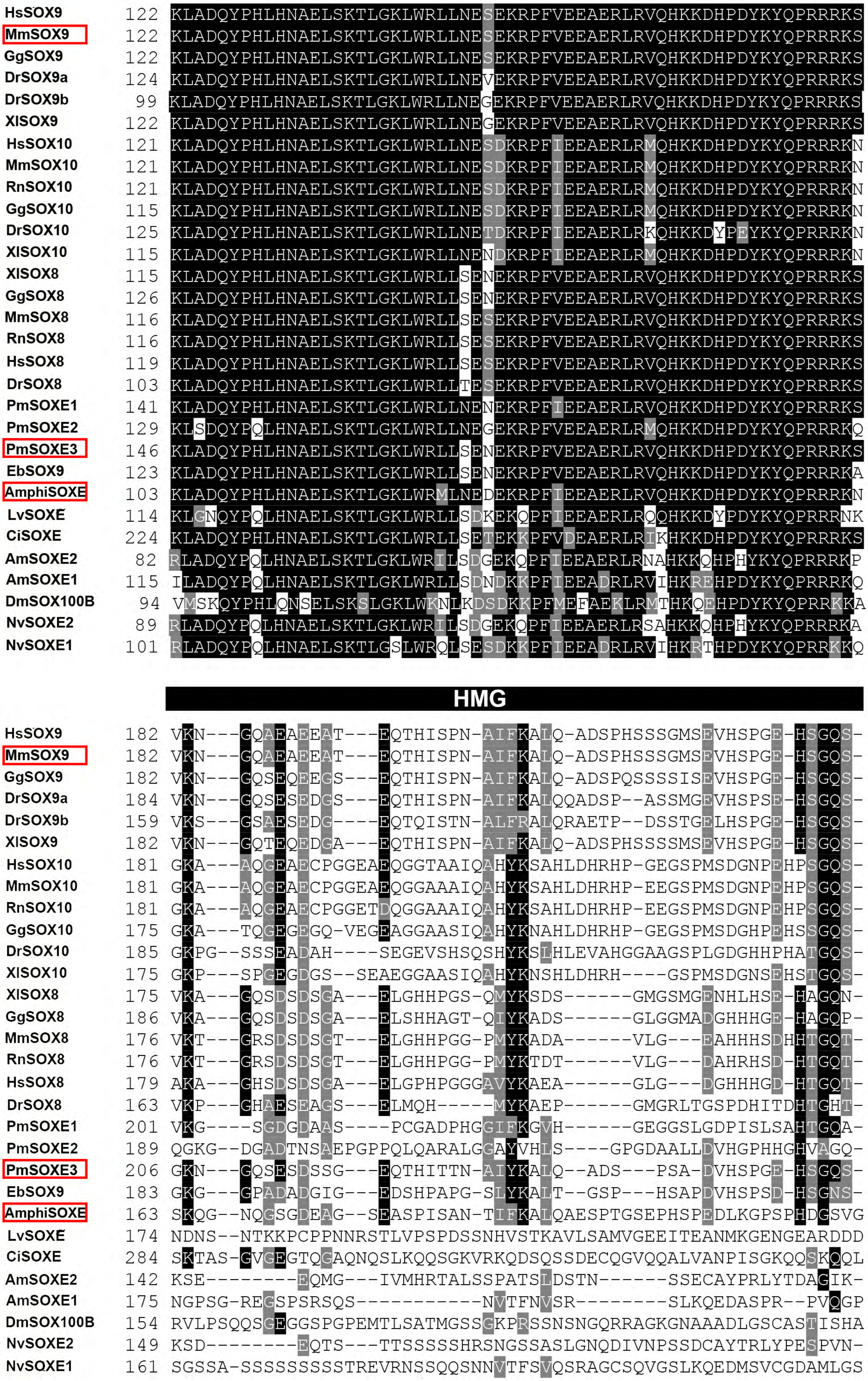

HsSOX9 122 KLADQYPHLHNAELSKTLGKLWRLLNESEKRPFVEEAERLRVQHKKDHPDYKYQPRRRKS
MmSOX9 122 KLADQYPHLHNAELSKTLGKLWRLLNESEKRPFVEEAERLRVQHKKDHPDYKYQPRRRKS
GgSOX9 122 KLADQYPHLHNAELSKTLGKLWRLLNESEKRPFVEEAERLRVQHKKDHPDYKYQPRRRKS
DrSOX9a 124 KLADQYPHLHNAELSKTLGKLWRLLNEVEKRPFVEEAERLRVQHKKDHPDYKYQPRRRKS
DrSOX9b 99 KLADQYPHLHNAELSKTLGKLWRLLNEGEKRPFVEEAERLRVQHKKDHPDYKYQPRRRKS
XlSOX9 122 KLADQYPHLHNAELSKTLGKLWRLLNEGEKRPFVEEAERLRVQHKKDHPDYKYQPRRRKS
HsSOX10 121 KLADQYPHLHNAELSKTLGKLWRLLNESDKRPFIEEAERLRMQHKKDHPDYKYQPRRRKN
MmSOX10 121 KLADQYPHLHNAELSKTLGKLWRLLNESDKRPFIEEAERLRMQHKKDHPDYKYQPRRRKN
RnSOX10 121 KLADQYPHLHNAELSKTLGKLWRLLNESDKRPFIEEAERLRMQHKKDHPDYKYQPRRRKN
GgSOX10 115 KLADQYPHLHNAELSKTLGKLWRLLNESDKRPFIEEAERLRMQHKKDHPDYKYQPRRRKN
DrSOX10 125 KLADQYPHLHNAELSKTLGKLWRLLNETDKRPFIEEAERLRKQHKKDYPEYKYQPRRRKN
XlSOX10 115 KLADQYPHLHNAELSKTLGKLWRLLNENDKRPFIEEAERLRMQHKKDHPDYKYQPRRRKN
XlSOX8 115 KLADQYPHLHNAELSKTLGKLWRLLSENEKRPFVEEAERLRVQHKKDHPDYKYQPRRRKS
GgSOX8 126 KLADQYPHLHNAELSKTLGKLWRLLSENEKRPFVEEAERLRVQHKKDHPDYKYQPRRRKS
MmSOX8 116 KLADQYPHLHNAELSKTLGKLWRLLSESEKRPFVEEAERLRVQHKKDHPDYKYQPRRRKS
RnSOX8 116 KLADQYPHLHNAELSKTLGKLWRLLSESEKRPFVEEAERLRVQHKKDHPDYKYQPRRRKS
HsSOX8 119 KLADQYPHLHNAELSKTLGKLWRLLSESEKRPFVEEAERLRVQHKKDHPDYKYQPRRRKS
DrSOX8 103 KLADQYPHLHNAELSKTLGKLWRLLTESEKRPFVEEAERLRVQHKKDHPDYKYQPRRRKS
PmSOXE1 141 KLADQYPHLHNAELSKTLGKLWRLLNENEKRPFIEEAERLRVQHKKDHPDYKYQPRRRKS
PmSOXE2 129 KLSDQYPQLHNAELSKTLGKLWRLLNEGEKRPFVEEAERLRMQHKKDHPDYKYQPRRRKQ
PmSOXE3 146 KLADQYPHLHNAELSKTLGKLWRLLSENEKRPFVEEAERLRVQHKKDHPDYKYQPRRRKS
EbSOX9 123 KLADQYPHLHNAELSKTLGKLWRLLSENEKRPFVEEAERLRVQHKKDHPDYKYQPRRRKA
AmphiSOXE 103 KLADQYPHLHNAELSKTLGKLWRMLNEDEKRPFIEEAERLRVQHKKDHPDYKYQPRRRKN
LvSOXE 114 KLGNQYPQLHNAELSKTLGKLWRLLSDKEKQPFIEEAERLRQQHKKDYPDYKYQPRRRNK
CiSOXE 224 KLADQYPHLHNAELSKTLGKLWRLLSETEKKPFVDEAERLRIKHKKDHPDYKYQPRRRKS
AmSOXE2 82 RLADQYPQLHNAELSKTLGKLWRILSDGEKQPFIEEAERLRNAHKKQHPHYKYQPRRRKP
AmSOXE1 115 ILADQYPQLHNAELSKTLGKLWRLLSDNDKKPFIEEADRLRVIHKREHPDYKYQPRRRKQ
DmSOX100B 94 VMSKQYPHLQNSELSKSLGKLWKNLKDSDKKPFMEFAEKLRMTHKQEHPDYKYQPRRKKA
NvSOXE2 89 RLADQYPQLHNAELSKTLGKLWRILSDGEKQPFIEEAERLRSAHKKQHPHYKYQPRRRKA
NvSOXE1 101 RLADQYPQLHNAELSKTLGSLWRQLSESDKKPFIEEADRLRVIHKRTHPDYKYQPRRKKQ
HMG
HsSOX9 182 VKN---GQAEAEEAT---EQTHISPN-AIFKALQ-ADSPHSSSGMSEVHSPGE-HSGQS-
MmSOX9 182 VKN---GQAEAEEAT---EQTHISPN-AIFKALQ-ADSPHSSSGMSEVHSPGE-HSGQS-
GgSOX9 182 VKN---GQSEQEEGS---EQTHISPN-AIFKALQ-ADSPQSSSSISEVHSPGE-HSGQS-
DrSOX9a 184 VKN---GQSESEDGS---EQTHISPN-AIFKALQQADSP--ASSMGEVHSPSE-HSGQS-
DrSOX9b 159 VKS---GSAESEDG----EQTQISTN-ALFRALQRAETP--DSSTGELHSPGE-HSGQS-
XlSOX9 182 VKN---GQTEQEDGA---EQTHISPN-AIFKALQ-ADSPHSSSSMSEVHSPGE-HSGQS-
HsSOX10 181 GKA---AQGEAECPGGEAEQGGTAAIQAHYKSAHLDHRHP-GEGSPMSDGNPEHPSGQS-
MmSOX10 181 GKA---AQGEAECPGGEAEQGGAAAIQAHYKSAHLDHRHP-EEGSPMSDGNPEHPSGQS-
RnSOX10 181 GKA---AQGEAECPGGETDQGGAAAIQAHYKSAHLDHRHP-EEGSPMSDGNPEHPSGQS-
GgSOX10 175 GKA---TQGEGEGQ-VEGEAGGAASIQAHYKNAHLDHRHP-GEGSPMSDGHPEHSSGQS-
DrSOX10 185 GKPG--SSSEADAH----SEGEVSHSQSHYKSLHLEVAHGGAAGSPLGDGHHPHATGQS-
XlSOX10 175 GKP---SPGEGDGS--SEAEGGAASIQAHYKNSHLDHRH----GSPMSDGNSEHSTGQS-
XlSOX8 175 VKA---GQSDSDSGA---ELGHHPGS-QMYKSDS------GMGSMGENHLHSE-HAGQN-
GgSOX8 186 VKA---GQSDSDSGA---ELSHHAGT-QIYKADS------GLGGMADGHHHGE-HAGQP-
MmSOX8 176 VKT---GRSDSDSGT---ELGHHPGG-PMYKADA------VLG---EAHHHSDHHTGQT-
RnSOX8 176 VKT---GRSDSDSGT---ELGHHPGG-PMYKTDT------VLG---DAHRHSD-HTGQT-
HsSOX8 179 AKA---GHSDSDSGA---ELGPHPGGGAVYKAEA------GLG---DGHHHGD-HTGQT-
DrSOX8 163 VKP---GHAESEAGS---ELMQH-----MYKAEP------GMGRLTGSPDHITDHTGHT-
PmSOXE1 201 VKG----SGDGDAAS----PCGADPHGGIFKGVH------GEGGSLGDPISLSAHTGQA-
PmSOXE2 189 QGKG--DGADTNSAEPGPPQLQARALGGAYVHLS----GPGDAALLDVHGPHHGHVAGQ-
PmSOXE3 206 GKN---GQSESDSSG---EQTHITTN-AIYKALQ--ADS---PSA-DVHSPGE-HSGQS-
EbSOX9 183 GKG---GPADADGIG---EDSHPAPG-SLYKALT--GSP---HSAPDVHSPSD-HSGNS-
AmphiSOXE 163 SKQG--NQGSGDEAG--SEASPISAN-TIFKALQAESPTGSEPHSPEDLKGPSPHDGSVG
LvSOXE 174 NDNS--NTKKPCPPNNRSTLVPSPDSSNHVSTKAVLSAMVGEEITEANMKGENGEARDDD
CiSOXE 284 SKTAS-GVGEGTQGAQNQSLKQQSGKVRKQDSQSSDECQGVQQALVANPISGKQQSKQQL
AmSOXE2 142 KSE--------EQMG---IVMHRTALSSPATSLDSTN------SSECAYPRLYTDAGIK-
AmSOXE1 175 NGPSG-REGSPSRSQS-----------NVTFNVSR--------SLKQEDASPR--PVQGP
DmSOX100B 154 RVLPSQQSGEGGSPGPEMTLSATMGSSGKPRSSNSNGQRRAGKGNAAADLGSCASTISHA
NvSOXE2 149 KSD--------EQTS---TTSSSSSHRSNGSSASLGNQDIVNPSSDCAYTRLYPESPVN-
NvSOXE1 161 SGSSA-SSSSSSSSSTREVRNSSQQSNNVTFSVQSRAGCSQVGSLKQEDMSVCGDAMLGS

```
HsSOX9     232 ---------QGPPTPPTTPKTDVQ-PG---KADLKREG---------------RPLPEGG
MmSOX9     232 ---------QGPPTPPTTPKTDVQ-AG---KVDLKREG---------------RPLAEGG
GgSOX9     232 ---------QGPPTPPTTPKTDAQQPG---KQDLKREG---------------RPLAEGG
DrSOX9a    233 ---------QGPPTPPTTPKTDTQ-PG---KADLKREA---------------RPLQENT
DrSOX9b    207 ---------QGPPTPPTTPKTDLPVCS---KADLKRER---------------ERDRERP
XlSOX9     232 ---------QGPPTPPTTPKTDIQ-PG---KPDLKREG---------------RPLQENG
HsSOX10    236 ---------HGPPTPPTTPKTELQSGKA----DPKRDG---------------RSMGEGG
MmSOX10    236 ---------HGPPTPPTTPKTELQSGKA----DPKRDG---------------RSLGEGG
RnSOX10    236 ---------HGPPTPPTTPKTELQSGKA----DPKRDG---------------RSLGEGG
GgSOX10    229 ---------HGPPTPPTTPKTELQAGKA----DSKREG---------------RSLGEGG
DrSOX10    238 ---------HSPPTPPTTPKTELQGGKSG---EGKREGGASRSGLGVGADGSSASSSASG
XlSOX10    225 ---------HGPPTPPTTPKTELQAGKS----DGKRDGS--------------HALREGG
XlSOX8     220 ---------HGPPTPPTTPKTDLHHG---GKQELKHEG---------------RRMMDNG
GgSOX8     231 ---------HGPPTPPTTPKTDLHHG---SKQELKHEG---------------RRLVESG
MmSOX8     219 ---------HGPPTPPTTPKTDLHQASNGSKQELRLEG---------------RRLVDSG
RnSOX8     218 ---------HGPPTPPTTPKTDLHQASNGSKQELRLEG---------------RRLVDSG
HsSOX8     222 ---------HGPPTPPTTPKTELQQA--GAKPELKLEG---------------RRPVDSG
DrSOX8     205 ---------HGPPTPPTTPKTEHPQAP---------------------------------
PmSOXE1    246 ---------QSPPTPPSTPKTEQGAKAGGGDAKRPLQAPESQTCVAPTAPSMGHSQQQQQ
PmSOXE2    242 --------PQSPPTPPNTPKTADHGPPGKGQGKRGHAGG-------------AAATGEGP
PmSOXE3    251 ---------QGPPTPPTTPKTDVQ-SN---KLDIKREG---------------RPLQEGG
EbSOX9     229 ---------QGPPTPPTTPKTEVH-GN---KLDIKREG---------------RPLQEGG
AmphiSOXE  218 VTPS----SQAPPTPPTTPKQDQGMTALK-ADGMKRDSTSNTLTAIHRDGPHHHHHHPQG
LvSOXE     232 GRGR----WTRTTYTPTTPKNDLDCTR-----PNKRQK---------------YSLKVKT
CiSOXE     343 SSHHSPQSVCHSPSNQSPPHQGSITNIYEVMHREQRGYHDSPDATVPCSPPTAINMDESK
AmSOXE2    184 -----------MYERPTYHDNKINYDVSR-------------------------------
AmSOXE1    213 NSPQSG----VSSSPPTTPNQGLSPPTP---PTTPRGQ---------------HYANQGN
DmSOX100B  214 N--------VGSNSSDVFSNEAFMKSLNSACAASLMEQSLIETGLDSPCSTASSMSSLTP
NvSOXE2    197 -----------KPMPVNTYGAELAPRVP--------------------------------
NvSOXE1    220 PQTTSGS--VGSSSPPTTPSQGLSPPTP---PTTPRGQ---------------HYVHQQH
```

```
HsSOX9     264 RQPP-------------------------------IDFRDVDIGELSSDVISNIETFDVN
MmSOX9     264 RQPP-------------------------------IDFRDVDIGELSSDVISNIETFDVN
GgSOX9     265 RQPPH------------------------------IDFRDVDIGELSSDVISNIETFDVN
DrSOX9a    265 GRPLS------------------------------INFQDVDIGELSSDVI---ETFDVN
DrSOX9b    240 LQDG-------------------------------IDFGAVDIGELSSDVISNIEAFDVN
XlSOX9     264 RQPPR------------------------------IDFRDVDIGELSSEVISTIEIFDVN
HsSOX10    268 KPH--------------------------------IDFGNVDIGEISHEVMSNMETFDVA
MmSOX10    268 KPH--------------------------------IDFGNVDIGEISHEVMSNMETFDVT
RnSOX10    268 KPH--------------------------------IDFGNVDIGEISHEVMSNMETFDVT
GgSOX10    261 KPH--------------------------------IDFGNVDIGEISHEVMSNMETFDVN
DrSOX10    286 KPH--------------------------------IDFGNVDIGEISHDVMANMEPFDVN
XlSOX10    258 KPQ--------------------------------IDFGNVDIGEISHDVMSNMETFDVN
XlSOX8     253 RQN--------------------------------IDFSNVDINELSSEVISNIEAFDVH
GgSOX8     264 RQN--------------------------------IDFSNVDISELSSEVINNMETFDVH
MmSOX8     255 RQN--------------------------------IDFSNVDISELSSEVISNMDTFDVH
RnSOX8     254 RQN--------------------------------IDFSNVDISELSSEVISNMDTFDVH
HsSOX8     256 RQN--------------------------------IDFSNVDISELSSEVMGTMDAFDVH
DrSOX8     223 KQN--------------------------------IDFSNVDISELSTDVIGNLT-FDLQ
PmSOXE1    297 HHQHSQQQQQHHNQLHHHHQQQQQQQAAATPARQHIDFSNVDMGELSSEVISNMEPFDVN
PmSOXE2    281 RHPS-------------------------------LDFQTIGMGDIAAEAISGMGNFDVN
PmSOXE3    283 RQQ--------------------------------IDFSNVDIRELSREVISNMESFDVN
EbSOX9     261 RQH--------------------------------IDFSNVDIRELSREVISNMESFDVN
AmphiSOXE  273 HGHPN------------------------------IDFSNVDIGPL--DVMSSMESFDVE
LvSOXE     268 EMP--------------------------------VDFAGVDVRDFGGDIMG-MEEFSSE
CiSOXE     403 KPHSTLHSR------NSSFGSKQQRGSSIDLTSECSSHSMHGVMDTNTQDIMAKPGFDVT
AmSOXE2    202 ------------------------------------SSWSNDTSKYPAD-HSIVESKGPY
AmSOXE1    251 QPQPSATMY--------------------------HHHQELGIGSSTEPSSQHQHQHQHQ
DmSOX100B  266 PATPYN---------------------------VAPSNAKASAANNPSLLIRQLSEPVAN
NvSOXE2    214 ----------------------------------------YEMAKYTIDQHGLTHGKCLE
NvSOXE1    260 HG---------------------------------SVYQQLDLGPNHMQASPEMVQHQQP
```

```
HsSOX9     293 EFDQYLPPNGHPGVPATHGQVT-YTGSYGIS------------------STAATPASAGH
MmSOX9     293 EFDQYLPPNGHPGVPATHGQVT-YTGSYGIS------------------STAPTPATAGH
GgSOX9     295 EFDQYLPPNGHPGVPATHGQVTTYSGTYGIS------------------SSASSPAGAGH
DrSOX9a    292 EFDQYLPPNGH--------QNAPYAGGY-------------------------------A
DrSOX9b    269 EFDQYLPPHGAPG-----PAGAGFSSGYG-----------------------------SA
XlSOX9     294 EFDQYLPPNGHPGVGST--QAS-YTGSYGIS------------------STPSATTGAGP
HsSOX10    296 ELDQYLPPN---GHPGH-------VSSYSAAG-------------YGLGSALAVASGH-S
MmSOX10    296 ELDQYLPPN---GHPGH-------VGSYSAAG-------------YGLGSALAVASGH-S
RnSOX10    296 ELDQYLPPN---GHPGH-------VGSYSAAG-------------YGLSSALAVASGH-S
GgSOX10    289 EFDQYLPPNGHAGHPGH-------VGGYAAAG-------------YGLGSALAAASGH-S
DrSOX10    314 ELDQYLPPN---GHPQASATAS--AGSAAPSYT------------YGISSALAAASGHST
XlSOX10    286 EFDQYLPPNGHAGHPSH-------IGGYTSSY--------------GLTGALAAGP---S
XlSOX8     281 EFDQYLPLNGHGAIPADHGQNT-TAAPYGPS----------------YPHAAG--ATPAP
GgSOX8     292 EFDQYLPLNGHTAMPADHGPG---AGFYSTS----------------YSHSAAGAGGAGQ
MmSOX8     283 EFDQYLPLNGHSALPTEPSQAT-ASGSYGGAS---------------YSHSGATGIGASP
RnSOX8     282 EFDQYLPLNGHSALATEPSQAT-ASGSYGGAS---------------YSHSGATGIGASP
HsSOX8     284 EFDQYLPLGG--PAPPEPGQA------YGGA----------------YFHAGAS-----P
DrSOX8     250 EFDQYLPLTP------DQGAC---------------------------------------
PmSOXE1    357 EFDQYLPHSQYGYGLTAAAVAAGWTAKLQEQE---------------RPTHIKTEQLSPS
PmSOXE2    310 EFDQYLPPSGHSSVIATNSAAAFAAGGFGGAVGGGPNASASSSSAAAAAATAASSLGQVA
PmSOXE3    311 EFDQYLPPNGHPG------HGQSVAASYGG----------------------TGYSINGH
EbSOX9     289 EFDQYRPPNGHPG------QGVYAPPSVYG----------------------LVGGGTPH
AmphiSOXE  301 EFDQYLPPNGHPASASGHHPSHPPYTSYSQMS-----------------SSATTTVTSSS
LvSOXE     295 ELDQYIVQTIASVTASQPMPCQQGMVRQTCAMP------PFTTHSSYPMSNVNTQSSNGR
CiSOXE     457 EFEQYMPGACNPAVVRQHEEAFGYSCMGEPHAKQKRCN--FTDTSQNMPSPVDCSVQQNP
AmSOXE2    225 EGGRYGESNVGKS------------------------------------CYVDAKCYETV
AmSOXE1    285 HQHQPGVDFR-YIEVGDGLP-IEEGQLNG-------------------LGSLAGVGLNLP
DmSOX100B  299 AGDGYGVLLEAGREYVAIGEVNYQGQSAGVQSGAEGGGAGQEMDFLENINGYGGYTGSRV
NvSOXE2    234 ATPRYADATA---------------------------------------HHHHHHHHHSL
NvSOXE1    287 EHQHSGVELPNYLELPDNVPTLEDEQLSN-------------------LGTIN---LGIP
```

```
HsSOX9     334 VWMSKQQAPPPPPQQPPQAPPAP--------------------------------QAPPQ
MmSOX9     334 VWMSKQQAPPPPPQQPPQAPQAP--------------------------------QAPPQ
GgSOX9     337 AWMAKQQPQPP---QPPAQPPA--------------------------------------
DrSOX9a    313 AWMTKPQNGSP-------------------------------------------------
DrSOX9b    295 AWMHKPLASSS-------------------------------------------------
XlSOX9     333 ACMSKQQQQQP-------------------------------------------------
HsSOX10    332 AWISKPPGVAL-------------------------------------------------
MmSOX10    332 AWISKPPGVAL-------------------------------------------------
RnSOX10    332 AWISKPPGVAL-------------------------------------------------
GgSOX10    329 AWISKQHGVSL-------------------------------------------------
DrSOX10    357 AWLSKQQ---L-------------------------------------------------
XlSOX10    322 AWALAK------------------------------------------------------
XlSOX8     322 VWSHKSSSTSS-------------------------------------------------
GgSOX8     333 VWTHKSPASAS-------------------------------------------------
MmSOX8     327 VWAHKGAPSAS-------------------------------------------------
RnSOX8     326 VWAHKGAPSAS-------------------------------------------------
HsSOX8     315 VWAHKSAPSAS-------------------------------------------------
DrSOX8     265 ------SRRAP-------------------------------------------------
PmSOXE1    402 HYSQQQQAAVQ-------------------------------------------------
PmSOXE2    370 AWAPAKSPVSGGD-----------------------------------------------
PmSOXE3    343 AWLSKQQQQQQQQ-----------------------------------------------
EbSOX9     321 TWLTPG------------------------------------------------------
AmphiSOXE  344 SWMAKQNTSPR-------------------------------------------------
LvSOXE     349 QWMGGRHHPSGGN-----------------------------------------------
CiSOXE     515 MFSPNKQGTPQYRPSSWVEGYETGVMTEASVSPNASMDQQFSQPENFSYDSVTSACSPLQ
AmSOXE2    249 KYHHEVSAAVK-------------------------------------------------
AmSOXE1    324 LNLQECEVESN-------------------------------------------------
DmSOX100B  359 SYPAYSYPANG-------------------------------------------------
NvSOXE2    255 KYPELQSKS---------------------------------------------------
NvSOXE1    325 LNLQECELE---------------------------------------------------
```

```
HsSOX9     362 PQAAPPQQPAAPPQQPQAHTLTTLSSEPGQSQ-RTHIKTEQL----SPSHY---------
MmSOX9     362 -QQAPPQQPQAP-QQQQAHTLTTLSSEPGQSQ-RTHIKTEQL----SPSHY---------
GgSOX9     356 -----------------QHTLPALSGEQGPAQQRPHIKTEQL----SPSHY---------
DrSOX9a    324 ----------------QSSQLTPLNPAEPDQPRTTHIKTEQL----SPSHY---------
DrSOX9b    306 -----------------------MANAGEQHQQRAQIKTEQL----SPGHY---------
XlSOX9     344 ----------------QQHSLSTLNSEQSQSQQRTHIKTEQL----SPSHY---------
HsSOX10    343 ----------------------PTVSPPGVD-AKAQVKTETAG-PQGPPHY---------
MmSOX10    343 ----------------------PTVSPPGVD-AKAQVKTETTG-PQGPPHY---------
RnSOX10    343 ----------------------PTVSPPAVD-AKAQVKTETTG-PQGPPHY---------
GgSOX10    340 ----------------------SATTSPVVD-SKAQVKTEGSA-PGG--HY---------
DrSOX10    365 ----------------------PSQQHLGADGGKTQIKSETHF-PGDT------------
XlSOX10    328 -----------------------QHSQTVAD-SKAQVKTESS----STSHY---------
XlSOX8     333 ------------------------SSSIESGQQRPHIKTEQL----SPSHY---------
GgSOX8     344 ------------------------PSSADSGQQRPHIKTEQL----SPSHY---------
MmSOX8     338 ------------------------ASPTEAGPLRPQIKTEQL----SPSHY---------
RnSOX8     337 ------------------------ASPTEAGPLRPHIKTEQL----SPSHY---------
HsSOX8     326 ------------------------ASPTETGPPRPHIKTEQP----SPGHY---------
DrSOX8     270 ------------------------PAGAHLHPQRVHIKTEQR----SPQHY---------
PmSOXE1    413 -------------------QQQQQNSPPPPQQQQQQQPTISYG-SFSIQHYGPPFSDLQN
PmSOXE2    383 -------------RERDGRERARGGGGGGGGGGGGGVKTEQQQQSVSPIRYSAHYQP-QQ
PmSOXE3    356 ------------QQQHTLSSPPPPPPAISSPEQRAHVKTEQL----SPSHY---------
EbSOX9     327 -------------TKTPLPAPSAPSSSPMNAEVRTHVKTEQL----SPSHY---------
AmphiSOXE  355 -------------------------DNAQEQRLPVKMEQEHLP-P-PPPQY---------
LvSOXE     362 ---------------------TSPLQATVLDNVNSKLEHDMMS---PPPQYP--------
CiSOXE     575 SSSVTSQSGEFNTYSMNQSSPHSCAELPVKKEQISPIQQHFFPHVPPATRFQCSELHKAA
AmSOXE2    260 -------------------------YHEAIP-KYSELQTKPYD----------------L
AmSOXE1    335 -----------------------ELDQYLRPQVPPPVHVPAPTTTTTASSS---------
DmSOX100B  370 ----------------------GHFATEEQQQQQALQASEALNYKPAAADID--------
NvSOXE2    264 -------------------------YDSPIPGTGTRYEPQKYP----------------D
NvSOXE1    334 --------------------------QYLPQQQS----LPALHQYGGQAIN---------
```

PQS

```
HsSOX9     408 --SEQQQ----------------------------HSPQQ-------IAYSPFN---LPH
MmSOX9     406 --SEQQQ----------------------------HSPQQ-------ISYSPFN---LPH
GgSOX9     386 --SEQQQ----------------------------HSPQQQQQQQQQLGYGSFN---LQH
DrSOX9a    355 --NEQQG-----------------------------SPQH-------ISYGSFNVQHLQH
DrSOX9b    330 --S-QQP------------------------------PQQ------------------QF
XlSOX9     375 --SDQQQQ---------------------------HSPQQ-------LNYSSFN---LQH
HsSOX10    370 --TDQPSTS-------------------------------------QIAYTSLS---LPH
MmSOX10    370 --TDQPSTS-------------------------------------QIAYTSLS---LPH
RnSOX10    370 --TDQPSTS-------------------------------------QIAYTSLS---LPH
GgSOX10    365 --TDQPSTS-------------------------------------QIAYTSLS---LPH
DrSOX10    390 ----AASGS-------------------------------------HVTYTPLT---LPH
XlSOX10    351 --TEQPSTS-------------------------------------QLTYTSLG---LPH
XlSOX8     356 --NDQSQG----------------------------SPTH-------SDYNTYS---AQA
GgSOX8     367 --SDQSHG----------------------------SPAH-------SDYGSYS---TQA
MmSOX8     361 --NDQSHG----------------------------SPGR-------ADYGSYS---AQA
RnSOX8     360 --NDQSHG----------------------------SPGR-------ADYGSYS---AQA
HsSOX8     349 --GDQPRG----------------------------SP----------DYGSCS---GQS
DrSOX8     293 --SEHS-------------------------------------------STLYS---SSS
PmSOXE1    453 ARSQQQQHYEYAEHHSGAHHHHHQQLQHHHGALHHHHHHQQQQQQQGSGHHQHSTAAVMS
PmSOXE2    429 QQQQQQQQQQQVGFGFQSHHPHHHHHHPHHHHHQQQHHHHQQQHQQQQAYAELQG-VQPP
PmSOXE3    391 --SDQQQQQQQPQQQQQHSPQQQQQQQQQQPQQAQQAQQQVQQQQQLGGYSPFS---IQH
EbSOX9     361 --SDTQAAQVQTQTGQQP-HQQQQQQQQQQSQQQQHSPPQSQQHPHLGNYIPFN---LQH
AmphiSOXE  379 --TPHPPAS---------------------------------------SYNYQP----QY
LvSOXE     390 --SSQQLHQ----------------------------------------MQAFHFAAMQQ
CiSOXE     635 DVAPQPYQAYFDANIQRAPMEDTMVDQRADALYHHNAYDHSGPFPNMHGRFDACSAKQQE
AmSOXE2    278 PK-------------------------------------------------------YPE
AmSOXE1    363 --SPWIFNR--------------------------------------YDEE-MERPSKRH
DmSOX100B  400 --PKEIDQY------------------------------------FMDQMLPMTQHHHPH
NvSOXE2    283 PKG------------------------------------------------------YPD
NvSOXE1    355 --SPWLSNR--------------------------------------YDDDCVERLGKRH
```

PQS

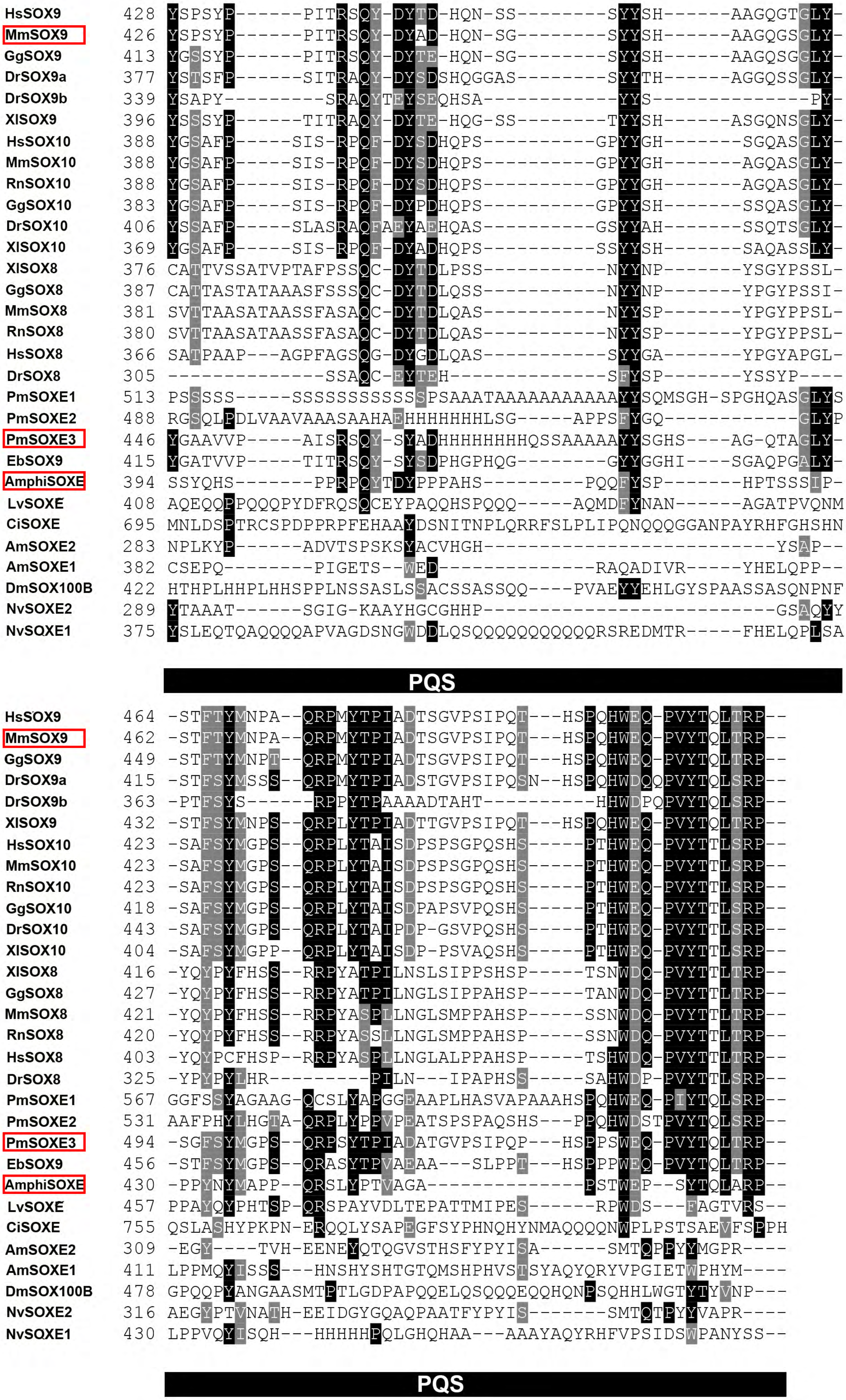
HsSOX9 428 YSPSYP------PITRSQY-DYTD-HQN-SS--------SYYSH------AAGQGTGLY-
MmSOX9 426 YSPSYP------PITRSQY-DYAD-HQN-SG--------SYYSH------AAGQGSGLY-
GgSOX9 413 YGSSYP------PITRSQY-DYTE-HQN-SG--------SYYSH------AAGQSGGLY-
DrSOX9a 377 YSTSFP------SITRAQY-DYSDSHQGGAS--------SYYTH------AGGQSSGLY-
DrSOX9b 339 YSAPY---------SRAQYTEYSEQHSA-------------YYS-------------PY-
XlSOX9 396 YSSSYP------TITRAQY-DYTE-HQG-SS--------TYYSH------ASGQNSGLY-
HsSOX10 388 YGSAFP-----SIS-RPQF-DYSDHQPS----------GPYYGH-------SGQASGLY-
MmSOX10 388 YGSAFP-----SIS-RPQF-DYSDHQPS----------GPYYGH-------AGQASGLY-
RnSOX10 388 YGSAFP-----SIS-RPQF-DYSDHQPS----------GPYYGH-------AGQASGLY-
GgSOX10 383 YGSAFP-----SIS-RPQF-DYPDHQPS----------GPYYSH-------SSQASGLY-
DrSOX10 406 YSSAFP-----SLASRAQFAEYAEHQAS----------GSYYAH-------SSQTSGLY-
XlSOX10 369 YGSAFP-----SIS-RPQF-DYADHQPS----------SSYYSH-------SAQASSLY-
XlSOX8 376 CATTVSSATVPTAFPSSQC-DYTDLPSS-----------NYYNP-------YSGYPSSL-
GgSOX8 387 CATTASTATAAASFSSSQC-DYTDLQSS-----------NYYNP-------YPGYPSSI-
MmSOX8 381 SVTTAASATAASSFASAQC-DYTDLQAS-----------NYYSP-------YPGYPPSL-
RnSOX8 380 SVTTAASATAASSFASAQC-DYTDLQAS-----------NYYSP-------YPGYPPSL-
HsSOX8 366 SATPAAP---AGPFAGSQG-DYGDLQAS-----------SYYGA-------YPGYAPGL-
DrSOX8 305 ---------------SSAQC-EYTEH--------------SFYSP-------YSSYP----
PmSOXE1 513 PSSSSS-----SSSSSSSSSSSPSAAATAAAAAAAAAAYYSQMSGH-SPGHQASGLYS
PmSOXE2 488 RGSQLPDLVAAVAAASAAHAEHHHHHHHLSG-----APPSFYGQ-----------GLYP
PmSOXE3 446 YGAAVVP-----AISRSQY-SYADHHHHHHHHQSSAAAAAYYSGHS----AG-QTAGLY-
EbSOX9 415 YGATVVP-----TITRSQY-SYSDPHGPHQG--------GYYGGHI----SGAQPGALY-
AmphiSOXE 394 SSYQHS-------PPRPQYTDYPPPAHS---------PQQFYSP-------HPTSSSIP-
LvSOXE 408 AQEQQPPQQQPYDFRQSQCEYPAQQHSPQQQ-----AQMDFYNAN------AGATPVQNM
CiSOXE 695 MNLDSPTRCSPDPPRPFEHAAYDSNITNPLQRRFSLPLIPQNQQQGGANPAYRHFGHSHN
AmSOXE2 283 NPLKYP------ADVTSPSKSYACVHGH-------------------------YSAP--
AmSOXE1 382 CSEPQ--------PIGETS--WED--------------RAQADIVR-----YHELQPP--
DmSOX100B 422 HTHPLHHPLHHSPPLNSSASLSSACSSASSQQ----PVAEYYEHLGYSPAASSASQNPNF
NvSOXE2 289 YTAAAT------SGIG-KAAYHGCGHHP-------------------------GSAQYY
NvSQXE1 375 YSLEQTQAQQQQAPVAGDSNGWDDLQSQQQQQQQQQQRSREDMTR-----FHELQPLSA
PQS
HsSOX9 464 -STFTYMNPA--QRPMYTPIADTSGVPSIPQT---HSPQHWEQ-PVYTQLTRP--
MmSOX9 462 -STFTYMNPA--QRPMYTPIADTSGVPSIPQT---HSPQHWEQ-PVYTQLTRP--
GgSOX9 449 -STFTYMNPT--QRPMYTPIADTSGVPSIPQT---HSPQHWEQ-PVYTQLTRP--
DrSOX9a 415 -STFSYMSSS--QRPMYTPIADSTGVPSIPQSN--HSPQHWDQQPVYTQLSRP--
DrSOX9b 363 -PTFSYS------RPPYTPAAAADTAHT-----------HHWDPQPVYTQLSRP--
XlSOX9 432 -STFSYMNPS--QRPLYTPIADTTGVPSIPQT---HSPQHWEQ-PVYTQLTRP--
HsSOX10 423 -SAFSYMGPS--QRPLYTAISDPSPSGPQSHS-----PTHWEQ-PVYTTLSRP--
MmSOX10 423 -SAFSYMGPS--QRPLYTAISDPSPSGPQSHS-----PTHWEQ-PVYTTLSRP--
RnSOX10 423 -SAFSYMGPS--QRPLYTAISDPSPSGPQSHS-----PTHWEQ-PVYTTLSRP--
GgSOX10 418 -SAFSYMGPS--QRPLYTAISDPAPSVPQSHS-----PTHWEQ-PVYTTLSRP--
DrSOX10 443 -SAFSYMGPS--QRPLYTAIPDP-GSVPQSHS-----PTHWEQ-PVYTTLSRP--
XlSOX10 404 -SAFSYMGPP--QRPLYTAISDP-PSVAQSHS-----PTHWEQ-PVYTTLSRP--
XlSOX8 416 -YQYPYFHSS--RRPYATPILNSLSIPPSHSP-----TSNWDQ-PVYTTLTRP--
GgSOX8 427 -YQYPYFHSS--RRPYATPILNGLSIPPAHSP-----TANWDQ-PVYTTLTRP--
MmSOX8 421 -YQYPYFHSS--RRPYASPLLNGLSMPPAHSP-----SSNWDQ-PVYTTLTRP--
RnSOX8 420 -YQYPYFHSS--RRPYASSLLNGLSMPPAHSP-----SSNWDQ-PVYTTLTRP--
HsSOX8 403 -YQYPCFHSP--RRPYASPLLNGLALPPAHSP-----TSHWDQ-PVYTTLTRP--
DrSOX8 325 -YPYPYLHR---------PILN---IPAPHSS-----SAHWDP-PVYTTLSRP--
PmSOXE1 567 GGFSSYAGAAG-QCSLYAPGGEAAPLHASVAPAAAHSPQHWEQ-PIYTQLSRP--
PmSOXE2 531 AAFPHYLHGTA-QRPLYPPVPEATSPSPAQSHS---PPQHWDSTPVYTQLSRP--
PmSOXE3 494 -SGFSYMGPS--QRPSYTPIADATGVPSIPQP---HSPPSWEQ-PVYTQLTRP--
EbSOX9 456 -STFSYMGPS--QRASYTPVAEAA---SLPPT---HSPPPWEQ-PVYTQLTRP--
AmphiSOXE 430 -PPYNYMAPP--QRSLYPTVAGA--------------PSTWEP--SYTQLARP--
LvSOXE 457 PPAYQYPHTSP-QRSPAYVDLTEPATTMIPES------RPWDS---FAGTVRS--
CiSOXE 755 QSLASHYPKPN-ERQQLYSAPEGFSYPHNQHYNMAQQQQNWPLPSTSAEVFSPPH
AmSOXE2 309 -EGY----TVH-EENEYQTQGVSTHSFYPYISA------SMTQPPYYMGPR----
AmSOXE1 411 LPPMQYISSS---HNSHYSHTGTQMSHPHVSTSYAQYQRYVPGIETWPHYM----
DmSOX100B 478 GPQQPYANGAASMTPTLGDPAPQQELQSQQQEQQHQNPSQHHLWGTYTYVNP---
NvSOXE2 316 AEGYPTVNATH-EEIDGYGQAQPAATFYPYIS-------SMTQTPYYVAPR----
NvSOXE1 430 LPPVQYISQH---HHHHHPQLGHQHAA---AAAYAQYRHFVPSIDSWPANYSS--
PQS

Supplemental Figure 1: Multiple Alignment of SOXE protein sequences

Alignment of SOXE protein sequences from *Homo sapiens* (Hs), *Mus musculus* (Mm), *Rattus norvegicus* (Rn), *Gallus gallus* (Gg), *Xenopus laevis* (Xl), *Danio rerio* (Dr), , *Petromyzon marinus* (Pm), *Eptatretus burgeri*(Eb), *Branchiostoma lanceolatum*(AmphiSOXE), *Ciona intestinalis* (Ci), *Lytechinus variegatus* (Lv), *Drosophila melanogaster* (Dm), *Apis mellifera* (Am), *Nasonia vitripennis* (Nv). by MEGA7[53] and is viewed by BoxShade.

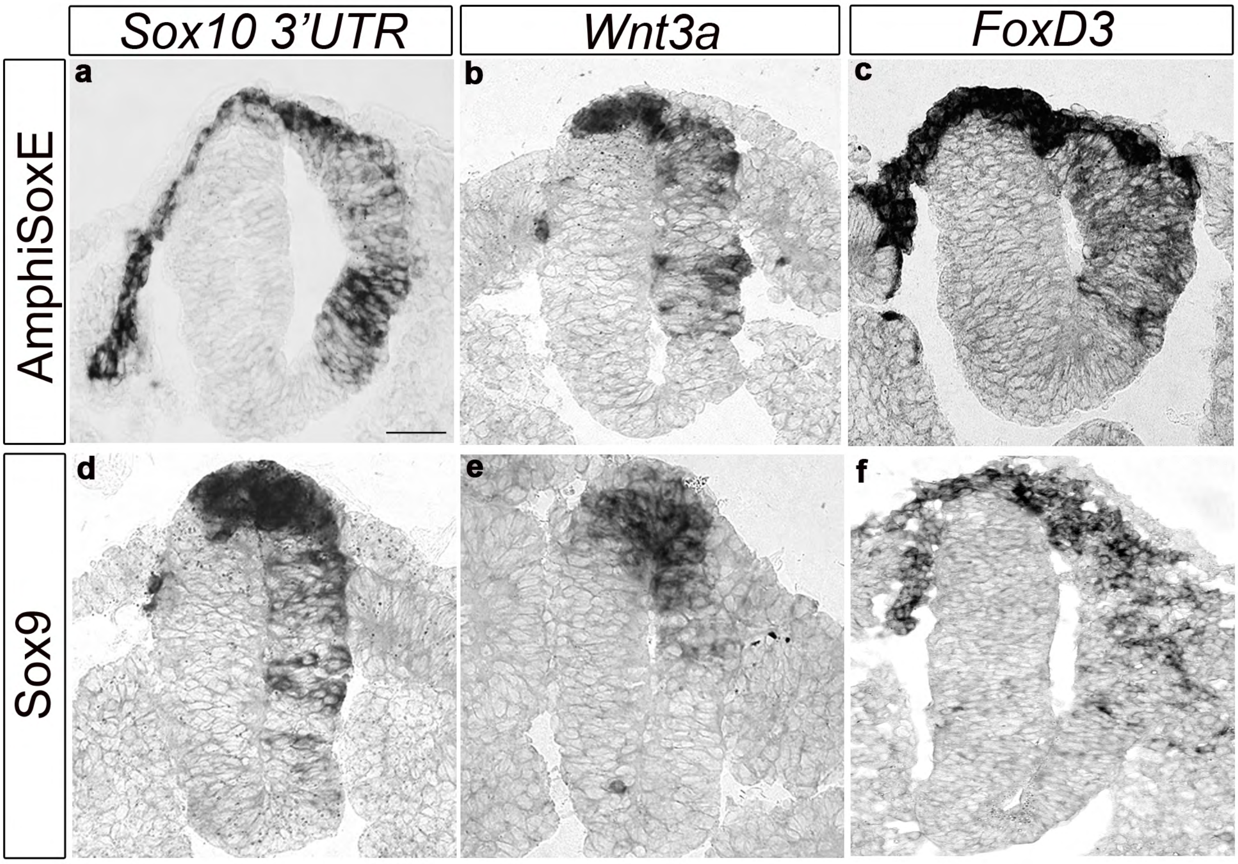

Supplemental Figure 2: Ectopic expression of AmphiSOXE in the chick neural tube induces neural crest markers

Transverse sections of the neural tube electroporated with *AmphiSoxE/EGFP* or *Sox9/EGFP* analysed at 24 hours post-transfection. Images are oriented with the transfected side to the right. (a,d) *Sox10*, (b,e) *Wnt3a* and (c,f) *FoxD3* (to a lesser extent) are induced to a similar degree by AmphiSOXE or SOX9. Scale bar: 100µm.

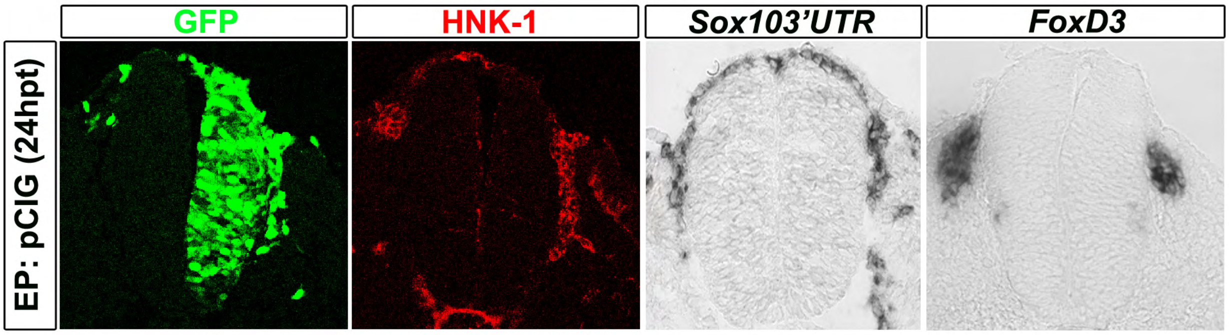

Supplemental Figure 3: Ectopic expression of pCIG control vector does not alter the expression of HNK1, *Sox10* and *Foxd3*

Transverse sections of the neural tube electroporated with pCIG vector and analysed at 24 hours post-transfection. Images are oriented with the transfected side to the right. Overexpression of pCIG does not induce ectopic or disrupt the endogenous expression of HNK1, *Sox10* and *FoxD3*.

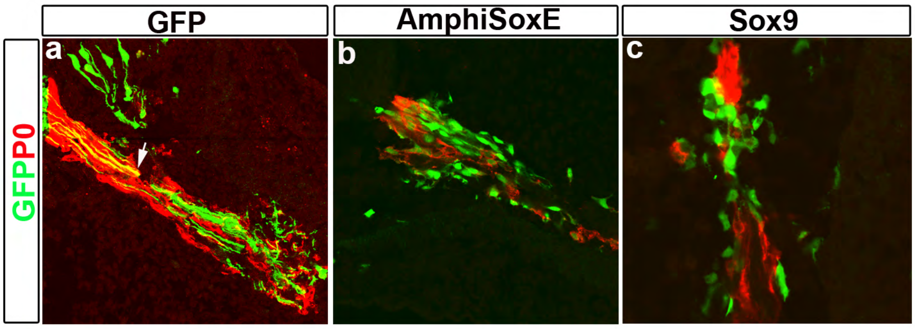

Supplemental Figure 4: Overexpression of AmphiSoxE or Sox9 affects Schwann cell differentiation

Transverse section of the neural tube electroporated with EGFP (a), *AmphiSoxE/EGFP* (b) and *Sox9/EGFP* (c) analyzed at 48 hours post-transfection. (a) Elongated EGFP+ cells line up with cells of the Schwann cell lineage marked by P0, expression of which appears to be downregulated by the ectopic expression of *AmphiSoxE* (b) or *Sox9* (c).

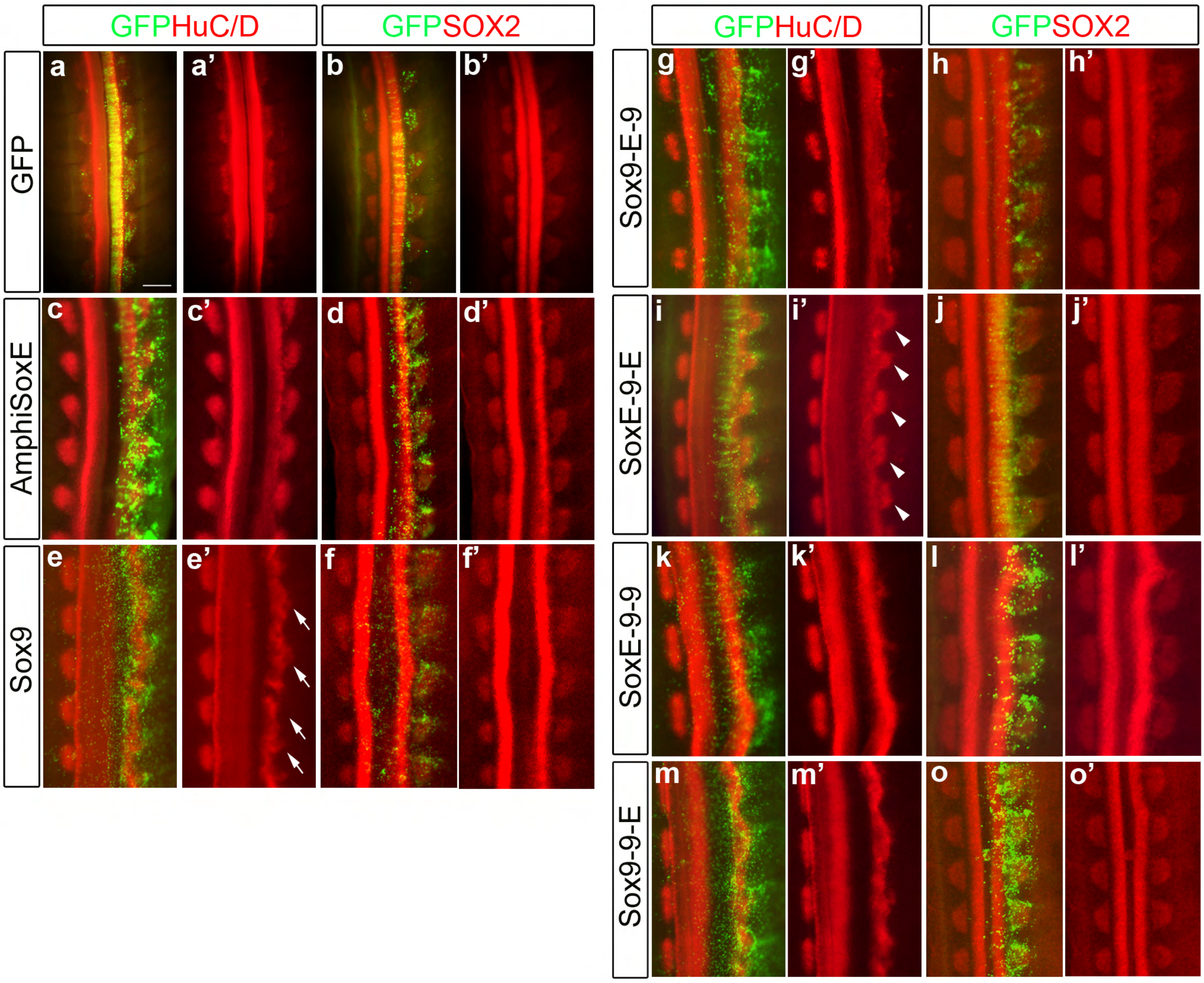

Supplemental Figure 5: Prolonged expression of chimeric protein harboring N- and C-domains of AmphiSOXE does not affect specification of neuron and glial lineages in the trunk

(a-o') Whole mount immunofluorescence of GFP/HuC/D and GFP/SOX2 on embryos electroporated with the constructs as indicated. Cells expressing GFP control (a-b'), AmphiSOXE (c-d') or SOXE-9-E (i-j') do not show altered expression of HuC/D and SOX2 in the DRG, whereas overexpression of SOX9 (e-f'), SOX9-E-9 (g-h'), SOXE-9-9 (k-l') or SOX9-9-E (m-o') downregulate HuC/D expression without affecting SOX2 protein expression. Scale bar: 20µm.

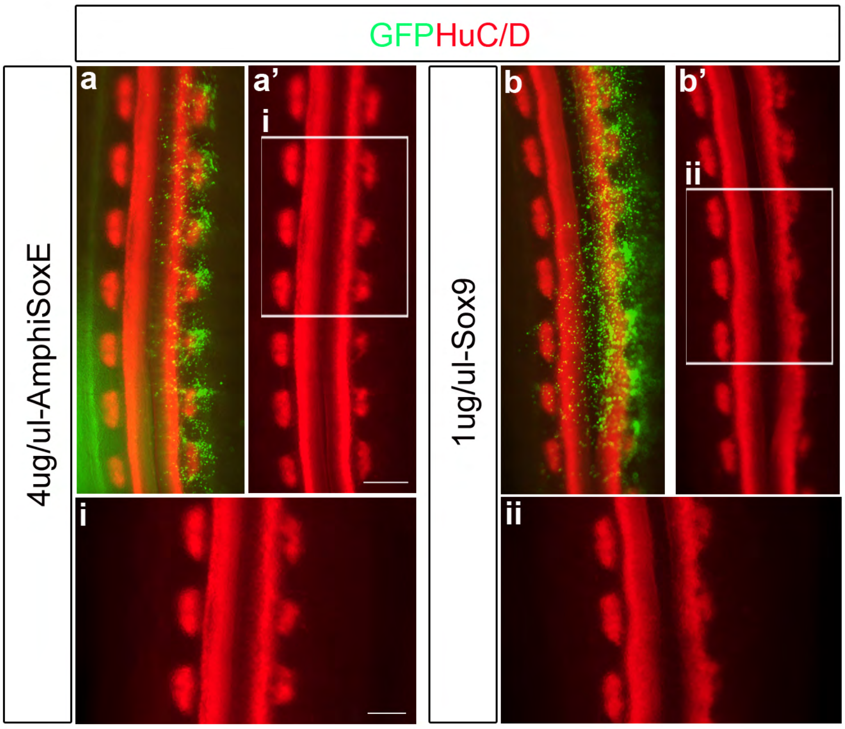

Supplemental Figure 6: Different concentrations of ectopic AmphiSOXE or SOX9 do not change HuC/D expression in the dorsal root ganglia

(a, a') HuC/D expression in the DRG is not significantly affected by increasing the concentration of ectopic *AmphiSoxE* expression to 4µg/µl, but reducing the ectopic *Sox9* expression to 1µg/µl still causes downregulation of HuC/D expression (b,b'). (i, ii) High-magnified view of region indicated by the white box in panels a', b'. Scale bars for A-b: 20µm, i-ii: 50µm.

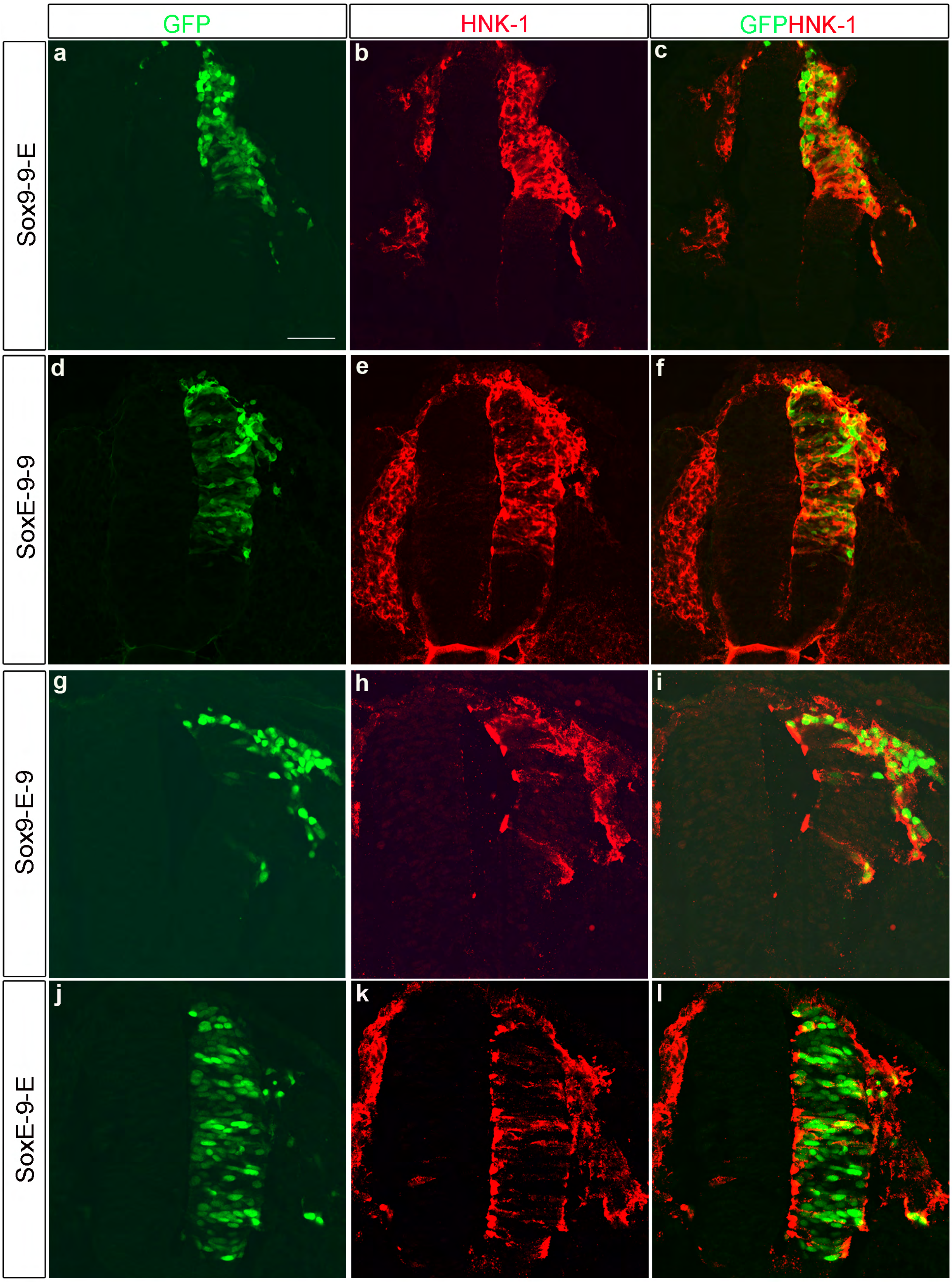

Supplemental Figure 7: Induction of neural crest markers by chimeric *SoxE* constructs

(a-l) SOX9-9-E, SOXE-9-9, SOX9-E-9 and SOXE-9-E can induce ectopic expression of HNK1. Scale bar: 100µm.

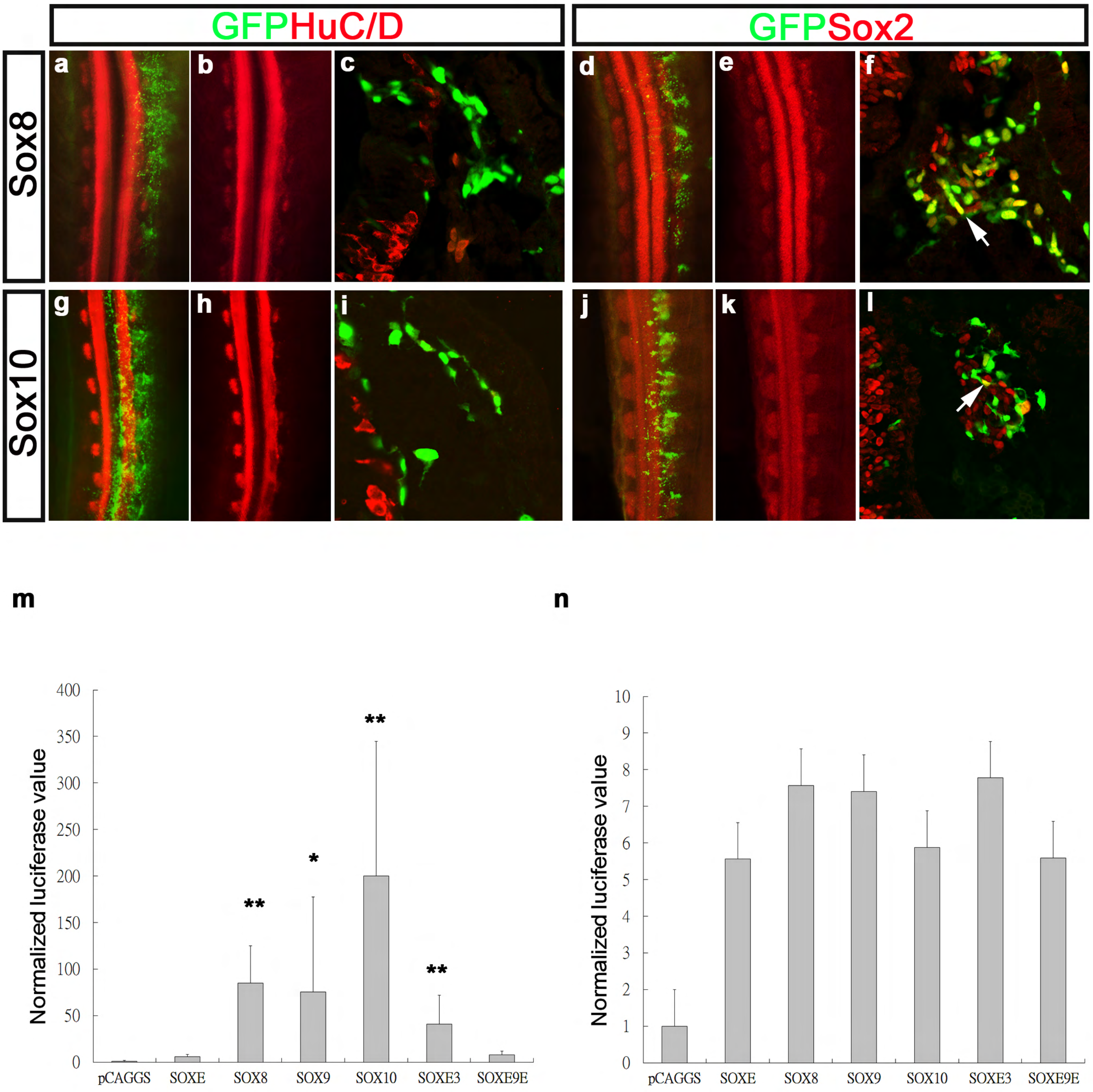
GFPHuC/D
GFPSox2
Sox8
Sox10
a
b
c
d
e
f
g
h
i
j
k
l
m
n
Normalized luciferase value
400
350
300
250
200
150
100
50
0
**
*
**
**
pCAGGS
SOXE
SOX8
SOX9
SOX10
SOXE3
SOXE9E
Normalized luciferase value
10
9
8
7
6
5
4
3
2
1
0
pCAGGS
SOXE
SOX8
SOX9
SOX10
SOXE3
SOXE9E

Supplemental Figure 8: Inhibition of neuronal fate by SOX8 and SOX10

HuC/D expression in DRG is lost in embryos transfected with *Sox8* (a-c) and *Sox10* (g-i). SOX2 expression in DRG is unaffected by ectopic expression of each construct (d-f, j-l). (m) In ovo *Sox2-NC1* driven luciferase reporter assays: *pCAGGS*, *AmphiSoxE*, *Sox8* ,*Sox9*, *Sox10*, *SoxE3* and *SoxE-9-E* were cotransfected into the neural tube with a *Sox2-NC1* luciferase reporter and a Renilla control plasmid in neural tube. The relative luciferase activity is compared with pCAGGS control. AmphiSOXE or SOXE-9-E protein weakly transactivates the *Sox2-NC1* luciferase reporter whereas SOX8, SOX9, SOX10 and SOXE3 yield exceptionally high and moderate activation of the reporter respectively. ** $p < 0.01$ and * $p<0.05$ as compared to AmphiSOXE (SOXE). (n) In ovo *Sox10-MCS4* driven luciferase reporter assays: *pCAGGS*, *AmphiSoxE*, *Sox8* ,*Sox9*, *Sox10*, *SoxE3* and *SoxE-9-E* were cotransfected into the neural tube with a *Sox10-MCS4* luciferase reporter and a Renilla control plasmid in neural tube. The relative luciferase activity is compared with pCAGGS control. All tested SOXE showed similar transactivation activity as no statistical difference was found.
